# Forward or Backward: Lessons Learned from Small Molecule Drugs Approved by FDA from 2012 to 2022

**DOI:** 10.3390/molecules28247941

**Published:** 2023-12-05

**Authors:** Mingxiao Gu, Sudan Sun, Qidong You, Lei Wang

**Affiliations:** 1 State Key Laboratory of Natural Medicines and Jiangsu Key Laboratory of Drug Design and Optimization, China Pharmaceutical University, Nanjing 210009, China; 2 Department of Medicinal Chemistry, School of Pharmacy, China Pharmaceutical University, Nanjing 210009, China

**Keywords:** new molecular entity, first-in-class, small-molecule drugs, physical and chemical properties

## Abstract

At every juncture in history, the design and identification of new drugs pose significant challenges. To gain valuable insights for future drug development, we conducted a detailed analysis of New Molecular Entitiy (NME) approved by the Food and Drug Administration (FDA) from 2012 to 2022 and focused on the analysis of first-in-class (FIC) small-molecules from a perspective of a medicinal chemist. We compared the change of numbers between all the FDA-approved NMEs and FIC, which could be more visual to analyze the changing trend of FIC. To get a more visual change of molecular physical properties, we computed the annual average trends in molecular weight for FIC across various therapeutic fields. Furthermore, we consolidated essential information into three comprehensive databases, which covered the indications, canonical SMILES, structural formula, research and development (R&D) institutions, molecular weight, calculated LogP (CLogP), and route of administration on all the small-molecule pharmaceutical. Through the analysis of the database of 11 years of approvals, we forecast the development trend of NME approval in the future.

## 1. Introduction

In recent years, there has been a significant increase in the approval of First-in-Class (FIC) drugs. Moreover, novel small-molecule drugs are emerging in therapeutic areas, such as oncology, immunology, and neurology. For example, Vitrakvi, a small-molecule drug that targets a specific genetic mutation, was approved by the Food and Administration (FDA) in 2018 for the treatment of various types of solid tumors [1]. Umbralisib, which has dual inhibition of phosphatidylinositol 3 kinaseδ (PI3Kδ) and casein kinase 1ε (CK1ε), has demonstrated significant clinical activity in patients with relapsed or refractory indolent non-Hodgkin lymphoma (MZL, FL, and SLL) [2]. However, as diverse approaches to drug discovery emerge in the future, many opportunities and challenges lie ahead for drug development. Capturing these opportunities will entail overcoming formidable scientific challenges.

Compared to traditional chemotherapy drugs, targeted therapeutic drugs have become mainstream cancer treatments due to their efficacy and safety. Targeted therapeutic drugs have rapidly developed all over the world because of the approval of the first small-molecule Tyrosine kinase inhibitor (TKI) Imatinib for clinical use by the FDA [3,4]. In recent years, there has been a significant increase in FDA-approved targeted drugs for cancer treatment. As targeted therapeutic drugs, small-molecule drugs have been increasingly used in cancer therapy to target specific pathways or molecules involved in cancer progression. Although there was such significant progress achieved, small-molecule-targeted anti-cancer drugs still face many challenges [4]. The major challenge is drug resistance, which was linked to many mechanisms, including gene mutation, amplification, Chronic sick cell syndrome (CSCS), apoptosis dysregulation, efflux transporters, and autophagy, etc. [5,6,7,8,9,10]. To deal with the challenges, there were many strategies applied, such as multitarget drugs, combination therapy, drugs targeting CSCS, and several new research trends [11,12]. Recent research has explored the potential of small-molecule drugs in immunotherapy, which is a type of cancer treatment that uses the body’s immune system to fight cancer [13]. There is a growing interest in small-molecule immuno-oncology drugs that target intracellular pathways [14]. Immuno-oncology (IO) is a transformative treatment option for cancer patients, marking one of the most recent advances in drug discovery and development in oncology [15]. Small-molecule drugs can expand the range of pathways that can be targeted and provide new target space for IO drug discovery [14]. At the same time, small-molecule drugs have the potential to be used as Immune checkpoint inhibitor (ICI) supplementary therapy [16]. Therefore, small-molecule drugs play a huge role in IO.

According to the World Health Organization (WHO), neurodegenerative diseases are projected to become the second leading cause of death after cardiovascular diseases, overtaking cancer by 2040 [17]. Despite heavy investment, treatment options for neurodegenerative diseases, including Amyotrophic lateral sclerosis (ALS), frontotemporal dementia, and Alzheimer’s disease, remain limited [18]. To this day, a disease-modifying therapy for Alzheimer’s disease remains elusive for Central nervous system (CNS) drug research. According to statistics, 190 investigational new drugs have failed in clinical trials [19]. In addition, according to a recent report by the Tufts Center for the Study of Drug Development, CNS drugs take 20% longer to develop and 38% longer to approve than non-CNS drugs [20]. Opioid addiction is another significant problem in central nervous system diseases. In the United States, opioid addiction has become an epidemic [21]. Recently, the first non-opioid drug for opioid withdrawal symptoms was approved. The drug, Lofexidine, is an α2a antagonist [22]. In a statement announcing the approval, the FDA reinforced its willingness to encourage and foster innovation, along with guidance to help accelerate approvals [23]. With the support and incentives from public agencies, more and more new CNS small-molecule drugs will be introduced to the market.

The 21st century has witnessed several viral epidemics and pandemics, including the severe acute respiratory syndrome (SARS) outbreak in 2002, the Zika virus epidemic from 2015–2016, the Ebola virus outbreak in West Africa from 2013–2016, and the yellow fever outbreak in 2016 [24]. However, compared to the inconceivable scale of the coronavirus disease 2019 (COVID-19) pandemic, caused by the severe acute respiratory syndrome coronavirus 2 (SARS-CoV-2), these outbreaks have been overshadowed. COVID-19, first identified in 2019, has led to high morbidity and mortality worldwide. As of 19 December 2021, COVID-19 has led to the death of over 5.3 million people worldwide [25]. To expedite the development of drugs and biologics (other than vaccines) for COVID-19 therapeutics, the FDA has created an emergency program, the Coronavirus Treatment Acceleration Program (CTAP) [26]. The first drug approved by the FDA for the treatment of COVID-19 was Remdesivir, an RNA polymerase inhibitor that was previously developed for the treatment of Ebola [27]. In addition to viral epidemics and pandemics, infectious tropical diseases also affect more than 1 billion people worldwide and continue to place a significant burden on developing countries. The Medicines for Malaria Venture (MMV) made significant progress in developing a pipeline for malaria medicines, which supported the development of Tafenoquine [28,29,30]. Tafenoquine is an investigational medicine, which was approved by the FDA in 2018 for the radical cure of Plasmodium vivax malaria [29]. In addition, with the increasing threat of bacterial resistance, the development of antibacterial drugs is more and more critical from a public health perspective. The WHO has given the highest priority to the development of drugs targeting carbapenem-resistant gram-negative bacteria [31]. According to a review, there were 47 direct-acting antibacterial drugs under clinical evaluation worldwide as of December 2022 [32].

Nowadays, cardiovascular diseases are also the number one cause of mortality worldwide, which claim almost 18 million lives annually [33]. Metabolic syndrome, characterized by conditions such as hypertension, hyperglycemia, dyslipidemia, and obesity, is a major driver of cardiovascular disease. Since the 1980s, the prevalence of obesity has increased in most countries worldwide [34]. According to the Global Burden of Disease (GBD) Obesity Collaborators, there were a total of 603.7 million adults with obesity in 2015. The prevalence of obesity doubled in 73 countries and continued to rise in most others between 1980 and 2015 [35]. With the increase in the obesity rate, the occurrence of cardiovascular diseases and metabolic syndrome is also gradually increasing all over the world [36].

In addition to the above major categories of diseases that threaten the health development of the world, there are a variety of other diseases. In recent years, the development of diseases tends to be more diversified and younger. The development trend of diseases has put forward new high requirements for drug research and development. However, drug research and development (R&D) is facing the problem of drug patent expiration and a large number of generic drugs [30,37,38,39,40,41]. The rise of the emerging biopharmaceutical industry is also having an impact on traditional chemical pharmaceuticals.

To gain valuable insights for future drug development, we conducted a detailed analysis of NME approved by the FDA from 2012 to 2022 and focused on the analysis of FIC small-molecule drugs from the perspective of medicinal chemists.

The FDA is committed to ensuring that the public has access to accurate information about the products it regulates, which plays a central role in drug development and patient care [42]. The drug development process consists of five steps: discovery and development, preclinical research, clinical research, FDA review, and FDA oost-market safety monitoring [43]. In this paper, small-molecule drugs are broadly defined as organic compounds with a molecular weight typically below 1000 Da. It is noteworthy that vancomycin, an antibiotic employed in the treatment of bacterial infections, possesses a molecular weight of approximately 1449 Da. This observation underscores the existence of small-molecule drugs with molecular weights surpassing the conventional threshold of 1000 Da.

According to the statistical results, there were only 10 new molecular entities (NMEs) approved by the FDA in 2016, of which 3 were FIC, and the number of pharmaceutical approvals was the lowest in nearly ten years [44]. In addition to these, the COVID-19 outbreak in 2019 has also harmed pharmaceutical R&D. The number of NMEs approved by the FDA in 2019 was 17, which is the second-lowest in nearly a decade. Taken together, these factors are forcing the traditional pharmaceutical industry to reinvent itself, stem the expected decline in productivity, and bring innovative new drugs to market. With the advent of modern molecular biology and the application of advanced technologies such as computer-aided drug design, structural biology, and combinatorial chemistry, small-molecule drugs have entered a phase of rapid development [45,46,47].

## 2. Multi-Angle Analysis of Small Molecule Drugs Approved by the FDA from 2012 to 2022

### 2.1. Analysis of Overall Trends in FDA Approval of NMEs

**Figure 1:**
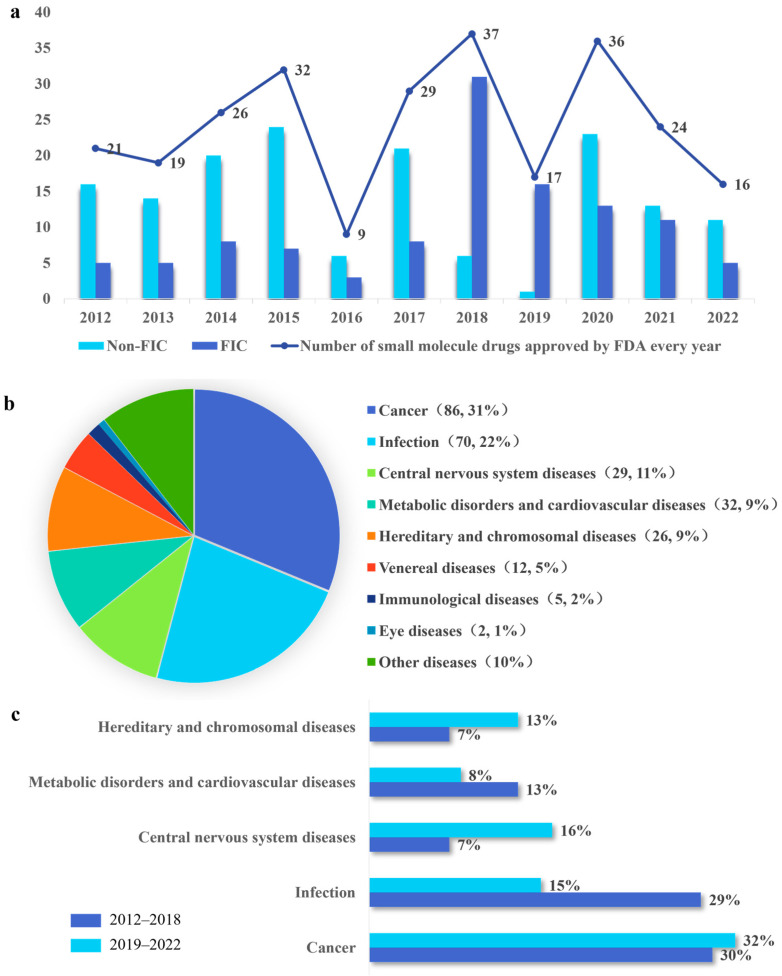
Overall trends in NMEs approved by the FDA from 2012 to 2022. (**a**) The trend of the number of NMEs approved by the FDA, and the number gap between FIC and non-FIC. (**b**) Analysis of all NMEs therapeutic areas approved by the FDA from 2012 to 2022. (**c**) Impact of COVID-19 on small-molecule drugs in different therapeutic areas. Therapy area approvals have been divided into two periods: 2012–2018 (Dark blue) and 2019–2022 (Baby blue). The research was based on the top four therapy areas for new approvals by the FDA from 2012 to 2022 to study the impact of the COVID-19 pandemic on drug development.

Annually, the global pharmaceutical industry innovates a diverse range of new drugs, offering significant medical advancements. The major categories include emerging biological drugs and traditional chemical small-molecule drugs [48]. Biologics are medicines derived from living cells or produced through biological processes, encompassing hormones, vaccines, blood products, and monoclonal antibodies [49]. Small molecules refer to drugs that are made by chemical synthesis, exemplified by compounds like aspirin, felbamate, and varenicline. Currently, while small-molecule drugs dominate the pharmaceutical market, biologics are rapidly gaining prominence. Biotechnology emerged as an innovative tool in medicine [50], giving rise to the development of the exciting drugs known as biologics. Recently, biologics have made significant strides in advancing healthcare. Nevertheless, small-molecule drugs remain to play a crucial role in innovative drug research and development. The current research and development of small-molecule drugs remains at a good pace. We examined and analyzed the small-molecule drugs approved by the FDA from 2012 to 2022. A total of 266 small-molecule drugs were approved by FDA (Figure 1) [51,52,53,54,55,56,57,58,59,60,61]. The median number of NME approvals per year was 25, with a low of 9 in 2016 and a high of 37 in 2018 during this time [44,62]. A possible explanation for this phenomenon is that fewer new drug applications were submitted in 2016, and the FDA rejected or delayed more drugs than in previous years. However, by focusing on rare diseases and orphan drugs and using rapid review programs and regulatory flexibility more often [57], the number of small-molecule drug approvals rose sharply from 2016 to 2018.

A FIC drug is a medication that treats a specific medical condition in a new and different way [63]. In, contrast, a non-FIC drug does not fall into the FIC category, potentially targeting molecules that have been targeted by preceding drugs or use a similar mechanism of action as existing drugs. FIC drugs are often referred to as innovative and provide new ways to help patients. From 2012 to 2022, there were 112 FICs, accounting for 42% of the total. Before 2018, there were fewer FIC drugs than non-FIC drugs. The proportion of FIC approvals reached its peak in 2018 and 2019, indicating that innovation and productivity across the pharmaceutical and biotech industries are on the rise. Despite a subsequent decline in FIC approvals post-2019, the level remained higher than that observed before 2018 (Figure 1a).

We comprehensively classify and analyze FDA-approved small-molecule drugs from the perspective of drug indications. The FDA has approved new small-molecule drugs across various therapy areas. We have divided them into nine therapeutic areas based on the types of indications. According to the analysis, from 2012 to 2022, the top five therapy areas for these new small-molecule drug approvals were cancer (31% of all approvals, 86), infection (22%, 70), CNS disorders (11%, 29), metabolic disorders and cardiovascular diseases (9%, 32), and hereditary and chromosomal diseases (26, 9%) (Figure 1b), which accounted for 83% of the total. FDA has had an expedited program for serious conditions since 1992, which allows accelerated approval, as well as other designations. Drugs were evaluated based on the patient’s need and evidence of clinical benefit [64]. It is expected that the R&D of drugs will be from the perspective of meeting the needs of patients. These initiatives have accelerated the pace of drug R&D.

The COVID-19 pandemic has exerted both positive and negative effects on drug R&D. On the positive side, it has stimulated the rapid development of vaccines and therapeutics for COVID-19, as well as the use of innovative technologies and platforms. On the negative side, it has disrupted the normal operations of clinical trials, regulatory agencies, and supply chains. Therefore, we analyzed the changes in the top five therapeutic areas of the NMEs approved from 2012 to 2022. To examine the possible impact of the COVID-19 pandemic on drug R&D, the analysis was split into two periods: 2012–2018 and 2019–2022. The number of drugs approved for CNS disorders rose from 7% of the total in 2012–2018 to 16% of the total in 2019–2022. Meanwhile, the number of drugs approved for hereditary and chromosomal diseases rose from 7% of the total in 2012–2018 to 13% of the total in 2019–2022. In contrast, the number of cancer approvals did not exhibit a marked trend, respectively. However, new drug approvals for infection, metabolic disorders, and cardiovascular diseases declined from 29% to 15%, and from 13% to 8% (Figure 1c).

In conclusion, the annual count of drugs approved by the FDA demonstrates a relatively stable pattern of fluctuation. While the total number of approvals has seen a slight decrease, the proportion of FIC drugs is consistently on the rise. In this decade, small-molecule drugs dedicated to treating cancer constitute a significant portion, reaching up to 31% of FDA approvals. Notably, the ongoing pandemic has not adversely impacted the development of drugs. The evolving disease landscape demands increased innovation in drug R&D. The discovery and development of FIC drugs are crucial for enhancing the productivity of the pharmaceutical industry [65]. The R&D of FIC drugs is essential for providing new therapeutic options to patients, particularly those with unmet medical needs. It also helps pharmaceutical enterprises diversify their portfolios, which is essential for their growth and sustainability. Therefore, To help more patients and sustain their business, pharmaceutical and biotech companies need to find a more effective way to produce novel drugs [66].

### 2.2. The Relationship between Pharmaceutical Enterprises or Academic Institutions and the Development of FIC Drugs

**Figure 2:**
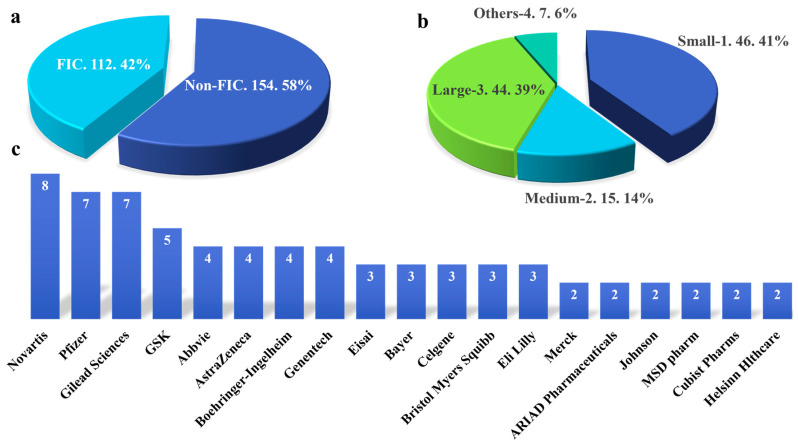
The relationship between FIC and R&D institutions. (**a**) The proportion of FIC in all small-molecule drugs approved by the FDA from 2012 to 2022. (**b**) The proportion of different institutions in FIC development (1 small biotech and small pharmaceutical enterprises, 2 medium pharmaceutical enterprises, 3 large pharmaceutical enterprises, 4 others including joint and academic). They were classified by company size. Pharmaceutical enterprises that have 1–1000 full-time employees at the time of R&D are considered small, 1000–10,000 employees—medium, and more than 10,000 employees—large. (**c**) The top 13 R&D institutions with the largest number of FIC small-molecule drugs.

Between 2012 and 2022, the FDA approved a total of 112 FIC drugs, accounting for 42% of all approved small-molecule drugs (Figure 2a). To investigate the contribution of various pharmaceutical companies to the R&D of FIC drugs, we compiled data from the R&D institutions of 112 FIC small-molecule drugs, categorizing them into four distinct groups. According to the number of full-time employees, we divided the pharmaceutical enterprises into three categories. Pharmaceutical enterprises that have 1–1000 full-time employees at the time of R&D are considered small. Others that have 1000–10,000 employees are considered medium. Pharmaceutical enterprises that have more than 10,000 employees are considered large. Small and medium enterprises (SMEs), especially young companies established in the 1990s and 2000s, developed 55% of all the FIC drugs. All SMEs that produced more than one FIC drug approved in 2019–2022 have demonstrated strong technological capabilities in a specific modality [67]. It is noteworthy that 41% of the FIC drugs were developed by small biotech and small pharmaceutical enterprises from 2012 to 2022 (Figure 2b). Therefore, small biotech and pharmaceutical enterprises continue to be the primary drivers of drug research and development in SMEs. Large pharmaceutical enterprises also play a crucial role in advancing medical research [68]. Large pharmaceuticals developed 39% of all the FIC drugs (Figure 2b). However, they are currently grappling with several challenges and tension. One of the primary challenges faced by big pharmaceutical companies is balancing the competing goals of efficiency and innovation [69]. Joint R&D could be a potential solution to address the challenges and tensions in drug discovery in large pharmaceutical enterprises [68]. Drug joint R&D refers to collaborative research and development efforts between multiple entities, such as pharmaceutical companies, academic institutions, and public institutions [70]. Collaborative research and development partnerships aim to pool resources, expertise, and knowledge to accelerate the discovery and development of new drugs. By working together, these entities can share the costs and risks associated with drug development, enhance productivity, and leverage each other’s strengths [71]. These reflect the importance of collaboration and strategic considerations in the pharmaceutical industry to improve drug discovery and development processes. According to our statistics, there are seven drugs developed through joint R&D between 2012 and 2022. Joint R&D has become a growing trend due to the increasing demand for drug research and development [67]. In addition, academic research institutions play a crucial role in drug discovery. They contribute to the development of innovative and FIC drugs by conducting basic research, identifying novel drug targets, and exploring new therapeutic modalities. These institutions often have access to cutting-edge technologies and expertise in specific areas of research, allowing them to make significant contributions to the field.

We further ranked companies according to the quantity of FDA-approved FIC drugs they developed. According to our statistics, Novartis developed the highest number of FIC drugs between 2012 and 2022. As a leading pharmaceutical company, it made significant contributions to the development of innovative drugs. From 2012 to 2022, Pfizer, Gilead Sciences, and GlaxoSmithKline (GSK), which are second-tier drug development companies, developed more than 5 FIC drugs. The statistical results above indicate that large pharmaceutical enterprises play a significant role in the development of FIC drugs. Abbvie, AstraZeneca, Boehringer-Ingelheim, Genentech, Eisai, Bayer, Celgene, Bristol Myers Squibb, and Eli Lilly developed three or four FIC drugs during that period. Merck, ARIAD Pharmaceuticals, Johnson & Johnson, MSD Pharmaceuticals, Cubist Pharmaceuticals, Helsinn Healthcare, and other drug development companies developed two FIC drugs each during that period (Figure 2c).

Therefore, the proportion of FIC drugs remains smaller than that of non-FIC drugs, and SMEs emerge as the primary driving force in the research and development of FIC. Currently, large pharma faces challenges in drug development, leading to an increasing trend in collaborative research and development initiatives. Novartis, as a key player in drug development, plays a significant role in the advancement of FIC drugs. While FIC drug R&D has broad prospects, there are still some challenges and difficulties. Among them, the problem of drug interaction and drug stability is one of the key problems to be solved in the development process. In the process of drug development and production, the chemical structure of drugs also has a significant impact on their stability, which needs to be adequately evaluated and controlled.

### 2.3. Trends in Molecular Weight Variation of FIC Small-Molecule Drugs

The fluctuations in molecular weight of chemical small-molecule drugs are both inevitable and necessary in the process of drug discovery and development. On one hand, these changes signify advancements and innovations in identifying new targets and mechanisms for treating various diseases. For instance, tazemetostat, a selective inhibitor of enhancer of zeste homolog 2 (EZH2), was approved by the FDA in 2020 [72], Remdesivir, a prodrug form of the monophosphate adenosine analog GS-441524, was approved in 2020 [73]. On the other hand, these changes also pose certain challenges and limitations about drug properties and performance. Increasing the molecular weight of small molecule drugs can reduce their solubility in water or biological fluids, which can affect their absorption, distribution, metabolism, and excretion (ADME) properties [74]. Therefore, small molecules are often preferred over larger molecules because they are easier to synthesize and have better pharmacokinetic properties. Small molecules can also be taken orally while others generally require injection or another form of parenteral administration.

**Figure 3:**
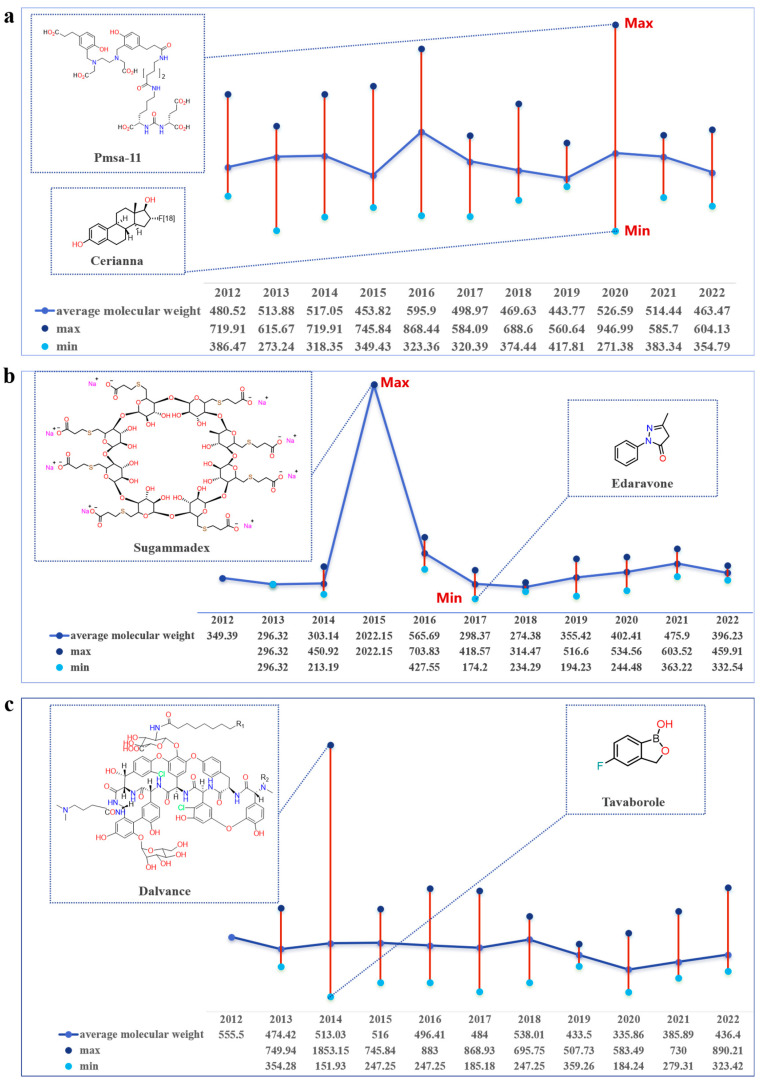
Average annual molecular weight changes of FIC drugs in different therapeutic fields. (cancer, central nervous system, infection). (**a**) Annual molecular weight changes of FIC drugs for cancer treatment. (**b**) Annual molecular weight changes of FIC drugs for central nervous system diseases. (**c**) Annual molecular weight changes of FIC drugs used to treat infection.

We calculated the molecular weights of small molecule drugs approved by the FDA in different therapeutic areas from 2012 to 2022 (Supplementary Table S1). At the same time, we also collated information about the route of administration and CLogP (Supplementary Table S2). This approach allows for the observation of how variations in molecular weight impact the physicochemical properties of the drug and its mode of administration. The drugs with the highest and lowest molecular weights were chosen, and their additional physical and chemical properties were compared and analyzed. Upon observation, it was noted that the annual average molecular weight of small molecule drugs in the field of cancer treatment exhibited minimal variation. The molecular weight changes from 2014 to 2017 showed a relatively obvious fluctuating trend (Figure 3a). The average molecular weight in 2015 was 453.82, while the average molecular weight in 2016 was 595.9. Venetoclax (Mr: 868.44), which was approved in 2016 for the treatment of leukemia, may be the reason for this change. Venetoclax has the highest molecular weight of the small molecule drugs approved by the FDA to treat cancer from 2012 to 2022 (Figure 3a); FDA approval has been granted for its use in treating chronic lymphocytic leukemia (CLL). This lipophilic molecule (CLogP: 10.10) is administered orally to treat various diseases. Cerianna is a PET imaging agent that detects estrogen-receptor-positive lesions in patients with recurrent or metastatic breast cancer [75]. As the drug with the lowest molecular weight, it exhibits high solubility and a rapid metabolic rate.

The analysis of data also unveiled substantial fluctuations in the molecular weight of small molecule drugs for CNS treatment from 2012 to 2022. Sugammadex, with a molecular weight of 2022, is the heaviest small molecule drug for infection treatment approved by the FDA from 2012 to 2022. Sugammadex is a modified γ-cyclodextrin with a lipophilic core and a hydrophilic periphery [76]. In addition, it is the first selective relaxant binding agent (SRBA) that reverses neuromuscular blockade induced by vecuronium bromide and rocuronium bromide, and its mechanism of action is superior to current neuromuscular blockade reversal strategies in terms of speed, efficacy, and side effects [77]. Sugammadex reverses the neuromuscular block caused by rocuronium bromide or vecuronium bromide by forming a complex with these drugs in the plasma, reducing the amount of neuromuscular blockers available to bind to nicotinic receptors in other neuromuscular junctions [78]. This unique mechanism makes Sugammadex a safer and more effective reversing agent than traditional reversing agents [79]. In adults with normal kidney function, the clearance of Sugammadex is estimated to be about 88 L/min, which is considered rapid. In addition, it has 100% bioavailability, does not bind to plasma proteins, and can be cleared by the kidney without modification [80]. Therefore, it can prevent the emergence of problems that are difficult to metabolize due to excessive molecular weight. Edaravone, which was approved by the FDA in 2017, is a free radical scavenger that can inhibit lipid peroxidation. This inhibition helps to prevent oxidative damage to brain cells, vascular endothelial cells, and nerve cells [81]. As the smallest molecular weight drug approved by the FDA for the treatment of the CNS system from 2012 to 2022, Edaravone is metabolized to produce two metabolites in the body, sulfate, and glycolaldehyde salts. Sulfate metabolites are the main metabolites and are excreted in the urine (Figure 3b).

The data analysis also disclosed a slight fluctuation in the molecular weight of small molecule drugs used to treat infections between 2012 and 2022. In 2014, the molecular weight of small molecule drugs reached its highest value, possibly due to the approval of Dalvance [82]. As a long-acting antimicrobial agent, it has excellent in vitro activity against gram-positive pathogens. In addition, the drug has a half-life ranging from 149 to 250 h in the human body. It has a long half-life of approximately 245 h, which allows for single-dose treatment, which reduces the need for hospitalization and improves treatment compliance [83]. Single-dose therapy reduces the risk of drug resistance and side effects. In addition to Vancomycin, Oritavancin exhibits rapid bactericidal activity against gram-positive bacteria, including methicillin-resistant Staphylococcus aureus (MRSA) [84]. Tavaborole, a topical antifungal drug for the treatment of onychomycosis, has the smallest molecular weight among the anti-infective drugs approved by the FDA from 2012 to 2022. As a small molecule with a low molecular weight, it easily penetrates the keratin fibers of the nails [85], and because it is water-soluble, this property helps it penetrate the nail bed. It is 40 times more effective at penetrating the nail bed than Ciclopirox [86]. The drug’s low molecular weight allows it to be widely metabolized by the human body, and it is primarily excreted through the kidneys after metabolism.

The molecular weight of a compound is a crucial physical property in drug development, influencing the metabolism, absorption, binding to targets, and overall properties and efficacy of the drug. According to the data results, as drug development progresses and targets are explored and researched, an increasing number of small molecule drugs with relatively high molecular weights are emerging. However, based on the analysis of the drugs with the highest and smallest molecular weight in cancer, CNS, and infection, it was observed that the increase in molecular weight did not significantly impact the ADME properties of the drugs in the body. It is important to characterize ADME for small-molecule drugs to reduce their failure rate because it provides critical information about the absorption, distribution, metabolism, and elimination properties of drug candidates. By gaining a deeper understanding of ADME properties, drug metabolism, and pharmacokinetics (DMPK), potential problems can be identified early in the drug discovery and development process [87,88,89,90]. This can ensure the accuracy and effectiveness of drug development.

We gathered and consolidated the structural formula, R&D institutions, and molecular weight of all small molecule compounds approved by the FDA from 2012 to 2022, which were summarized in Supplementary Table S1. We also compiled information on indications, CLogP, and route of administration, summarizing it in Supplementary Table S1, and canonical SMILES in Supplementary Table S2.

## 3. Conclusions

We conducted a comprehensive analysis of NMEs approved by the FDA from 2012 to 2022, examining trends in quantity, R&D institutions, treatment types, and fundamental physical and chemical properties. This assessment allowed us to identify patterns in drug R&D and make predictions regarding future development trends. In conclusion, the evolving disease landscape necessitates increased innovation in drug development. The identification and development of FIC drugs play a pivotal role in enhancing the pharmaceutical industry’s productivity. However, despite the broad prospects of FIC drug development, challenges and difficulties persist. Notably, problems such as drug interaction and stability pose key challenges to be addressed in the development process. In the process of drug development and production, the chemical structure of drugs significantly impacts their stability during development and production, necessitating thorough evaluation and control. The molecular weight of a compound is an important physical property in drug development, influencing the metabolism and absorption of the drug, the binding of the drug to the target, and the properties and efficacy of the drug. Therefore, we calculated the molecular weight and other physicochemical properties of small molecule drugs. Statistics show that more and more small-molecule drugs with relatively high molecular weight are appearing. However, it was found that the increase in molecular weight did not affect the ADME properties of the drugs in the body. Presently, the research and development of small-molecule drugs face significant challenges. Nevertheless, the rapid development of small-molecule drugs has been propelled by the advent of modern molecular biology and the application of advanced technologies, including computer-aided drug design, structural biology, and combinatorial chemistry.

## Data Availability

The data is mainly sourced from the FDA website U.S. Food and Drug Administration (fda.gov). The molecular weight and CLogP information for all small molecular compounds was calculated with ChemBioDraw Ultra 14.0. The Canonical SMILES information for all small molecular compounds was retrieved using PubChem.

## Supplementary Materials

The following supporting information can be downloaded at: https://www.mdpi.com/article/10.3390/molecules28247941/s1.
