# Supplementary material for: Forward or Backward: Lessons Learned from Small Molecule Drugs Approved by FDA from 2012 to 2022
Source: Molecules. 2023 Dec 5;28(24):7941. doi: 10.3390/molecules28247941 (PMC10745639; doi:10.3390/molecules28247941)
Supplement: Supplementary file 1 [file molecules-28-07941-s001.zip › molecules-2705409-supplementary.pdf]

Supplementary Table S1. The structural formula, R&D institutions, and Molecular administration of small molecule drugs approved by the FDA between 2012 and 2022.

Drug name 2012	Structural formula	R&D institutions	Molecular Weight (Da)
Orkambi	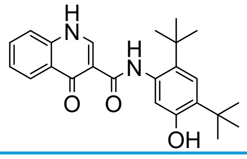	Vertex	392.49
Xeljanz	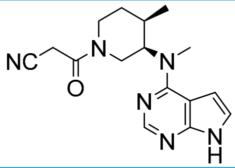	Pfizer	312.37
Xtandi	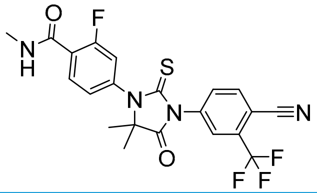	Astellas	464.44
Eliquis	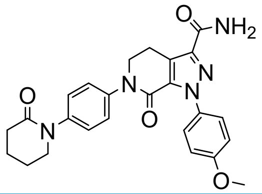	Pfizer &Bristol Myers Squibb	459.50
Picato	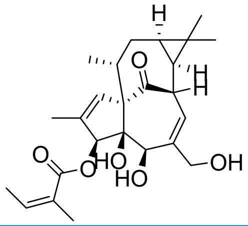	LEO Pharma AS	430.53
Inlyta	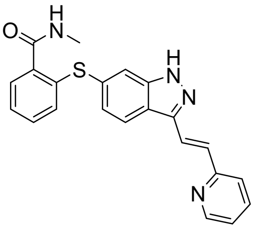	Pfizer Inc	386.47
Erivedge	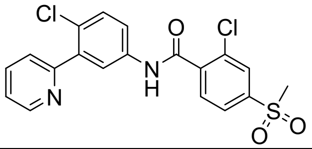	Genentech Inc	421.30

Zioptan	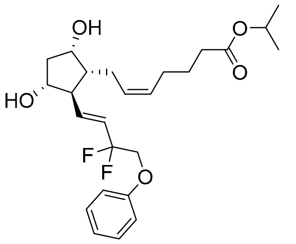	Merck	452.53
Stendra	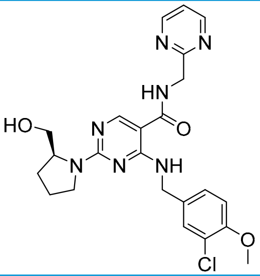	Vivus	483.95
Belviq	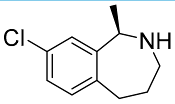	Eisai	195.08
Mybetriq	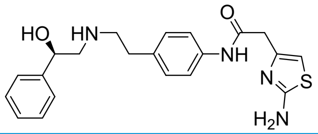	AstraZeneca	396.5
Kyprolis	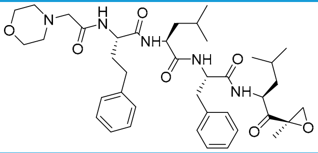	Onyx	719.91
Tudorza Pressair	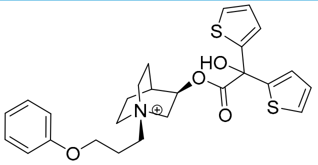	Forest Pharmaceuticals	564.55
Bosulif	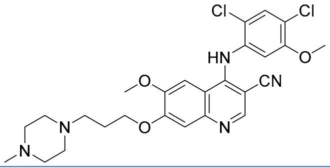	Pfizer	530.45
Aubagio	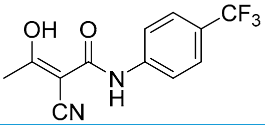	Sanofi	270.21
Stivaga	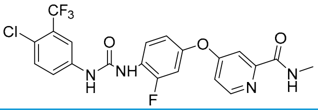	Bayer	482.82
Fycompa	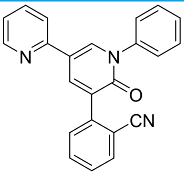	Eisai	349.12

Cometriq	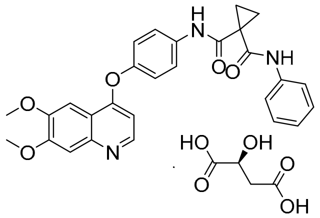	Exelixis	501.51
Lclusig	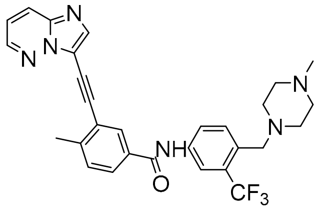	ARIAD Pharmaceuticals	532.56
Juxtapid	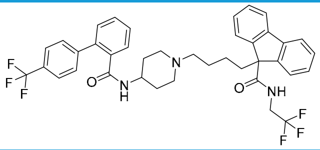	Aegerion	693.72
Sirturo	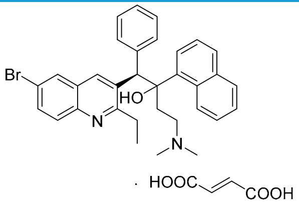	Johnson	555.50

**Drug name 2013**	**Structural formula**	**R&D institutions**	**Molecular Weight (Da)**
Pomalyst	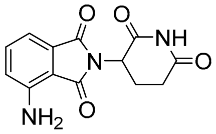	Celgene	273.24
Tafinlar	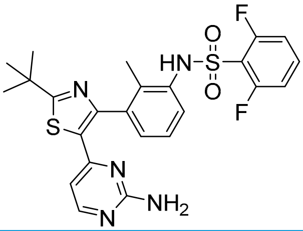	GSK	615.67
Mekinist	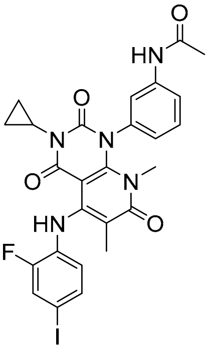	GSK	615.19
Gilotrif	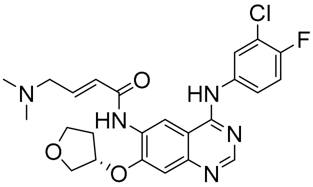	Boehringer-Ingelheim	485.94

Imbruvica	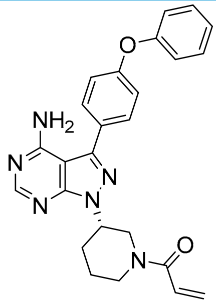	Pharmacyclic & Johnson	440.50
Tivicay	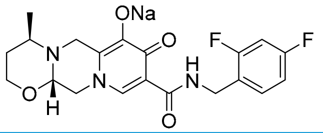	Viiv Healthcare	419.38
Olysio	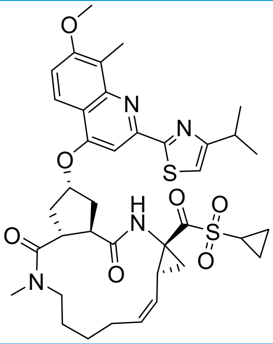	Johnson	749.94
Sovaldi	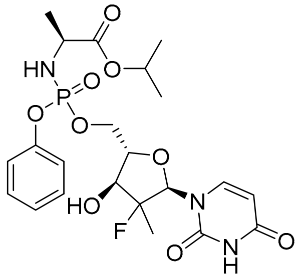	Gilead	529.45
Nesina	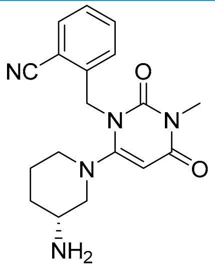	Takeda	461.51
Invokana	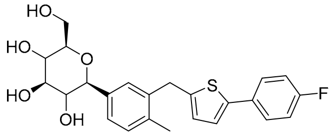	Johnson	444.52
Adempas	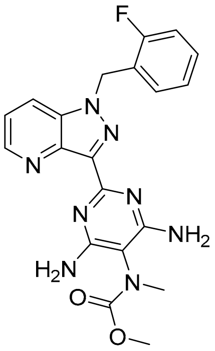	Bayer	422.42

Opsumit	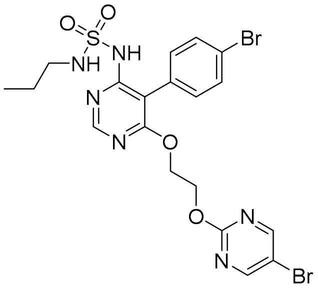	Actelion Pharms Ltd	588.27
Breo Ellipta	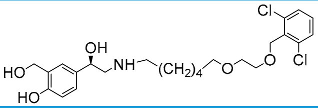	GSK	774.77
Bromide	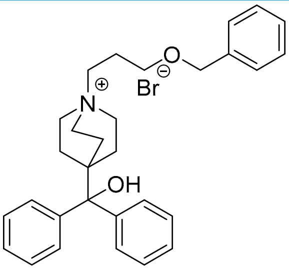	GSK	508.49
Osphena	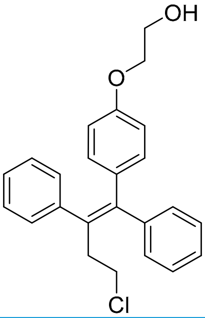	SHIONOGI	378.89
Tecfidera	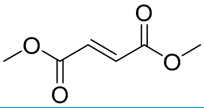	Biogen Idec	144.13
Brintellix	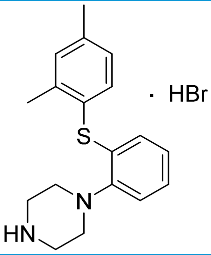	Takeda & Lundbeck	298.45
Aotiom	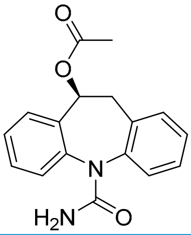	Sunovion	296.32
Luzu	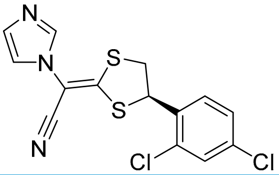	Japan Pesticide Corporation	354.28
**Drug name**	**Structural formula**	**R&D**	**Molecular Weight (Da)**

2014		institutions	
Zykadia	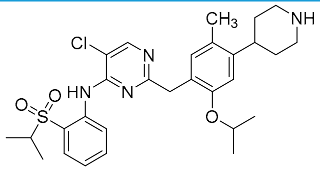	Novartis	557.15
Beleodaq	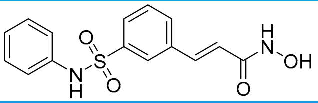	Spectrum Pharms	318.35
Zydelig	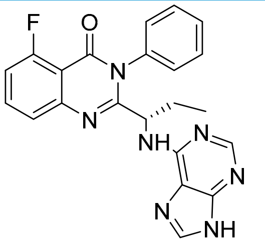	Gilead Sciences	415.42
Ledipasvir,	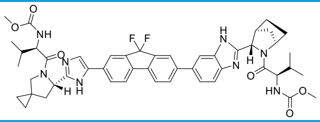	Gilead Sciences	889.00
Rapivab	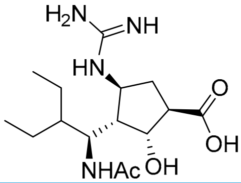	BioCryst Pharmaceuticals	328.41
Viekira Pak	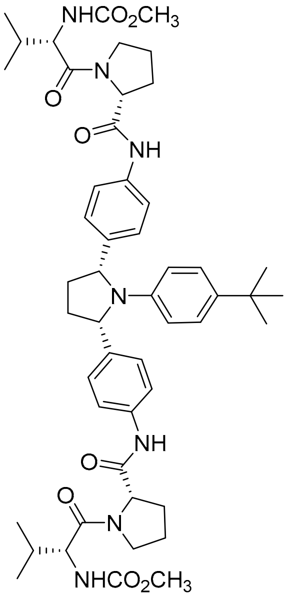 Ombitasvir	Abbive	894.11
	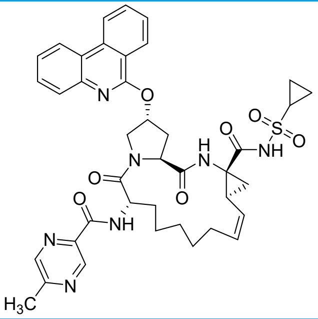		765.88

	Paritaprevir		
	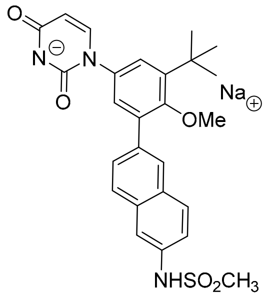 Dasabuvir		493.57
Impavido	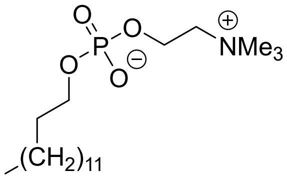	Paladin Therapeutics	407.57
Farxiga	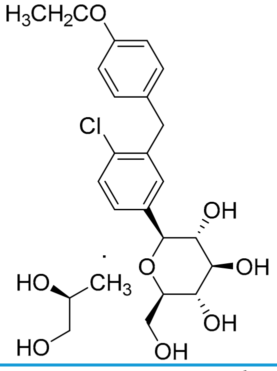	AstraZeneca & Bristol Myers Squibb	408.87
Jardiance	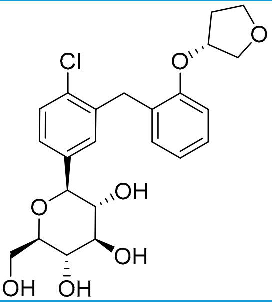	Boehringer -Ingelheim	450.91
Northera	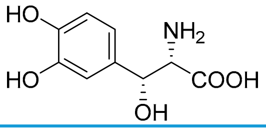	Lundbeck NA	213.19
Hetlioz	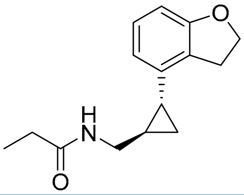	Vanda Pharms	245.32

Belsomra	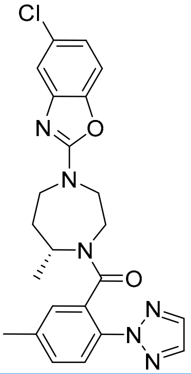	MSD pharm	450.92
Cerdelga	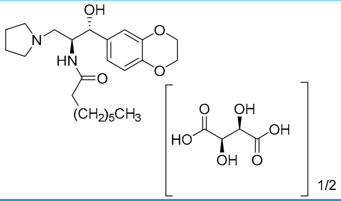	Genzyme Corporation	404.54
Movantik	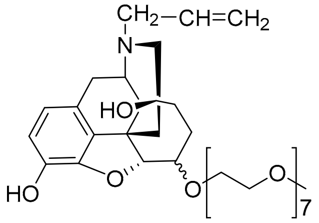	AstraZeneca	403.52
Striverdi Respimat	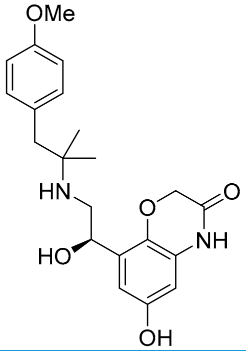	Boehringer -Ingelheim	422.90
Sivextro	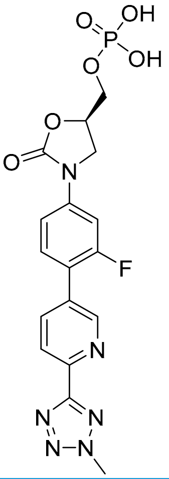	Cubist Pharms	450.32

Xtoro	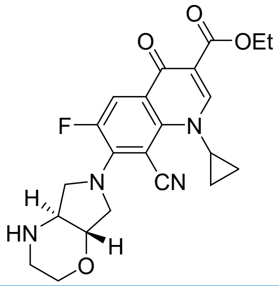	Alcon Res Ltd	398.39
Zerbaxa	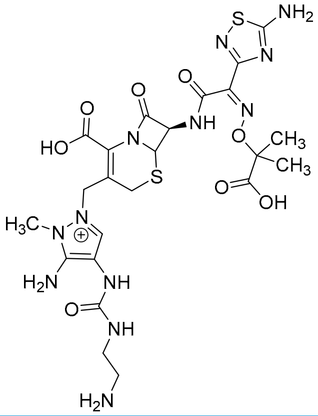	Cubist Pharms	667.70
Dalvance	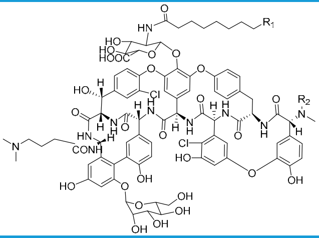	Durata Theraps	1853.15
Jublia	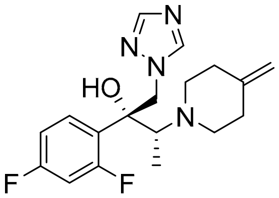	Kaken Pharmaceutical CO., LTD & Valeant	348.39
Kerydin	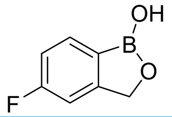	Anacor Pharms	151.93
Otezla	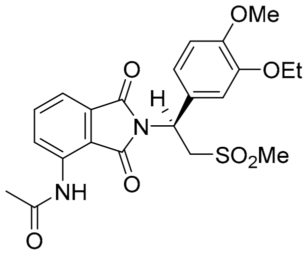	Celgene Corp	460.50

Zontivity	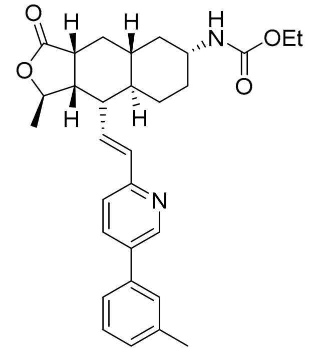	MSD	590.66
Akynzeo	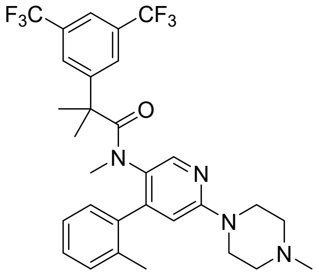	Helsinn Hlthcare	578.59
Esbriet	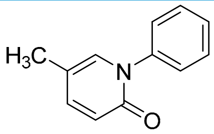	InterMune	185.22
Ofev	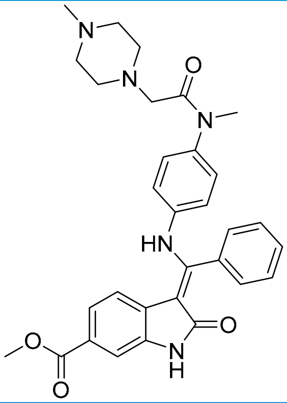	Boehringer-Ingelheim	539.62
**Drug name 2015**	**Structural formula**	**R&D institutions**	**Molecular Weight (Da)**
Ibrance	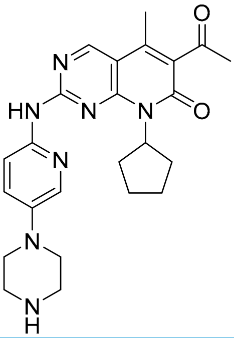	Pfizer Inc.	447.53

Lenvima	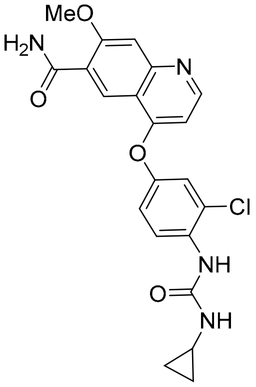	Eisai	426.85
Fardak	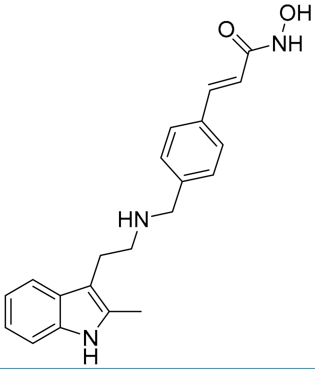	Novartis	349.43
Odomzo	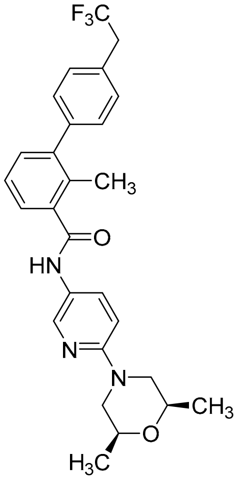	Novartis	485.50
Lonsurf	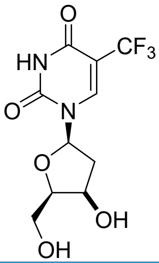	Taiho cancerlnc	296.20
Cotellic	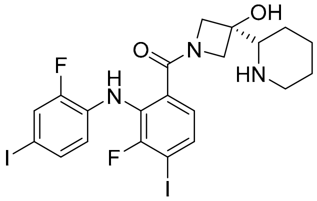	Genentech	531.31

Ninlaro	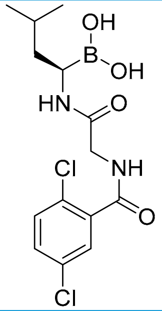	Millennium pharmaceutical	361.03
Yonndelis	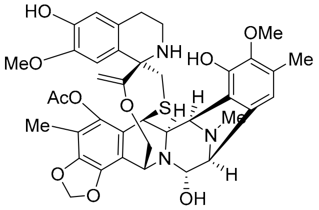	PharmaMar	745.84
Farydak	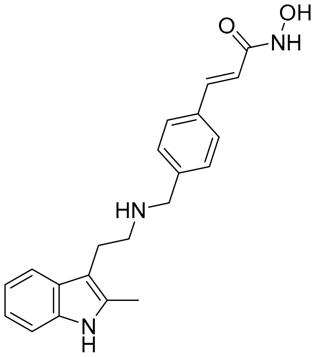	Novartis	349.43
Tagrisso	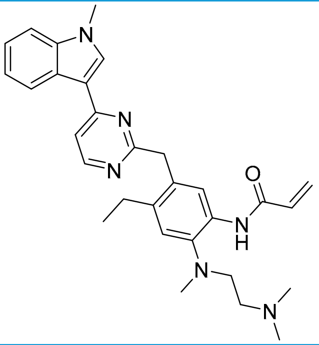	AstraZeneca	499.61
Alecensa	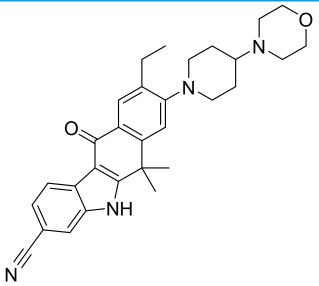	Chugai pharmaceutical	482.62

Corlanor	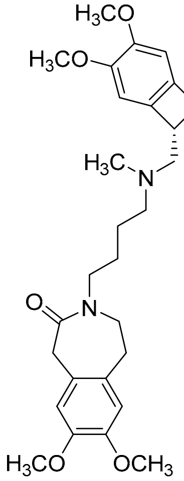	Servier	468.59
Savaysa	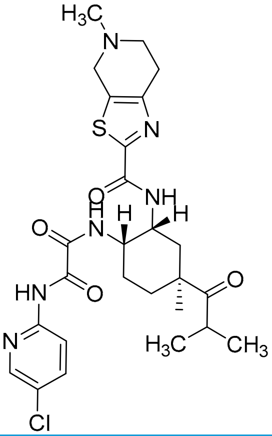	Daiichi Sankyo Company Limited	548.06
Kengreal	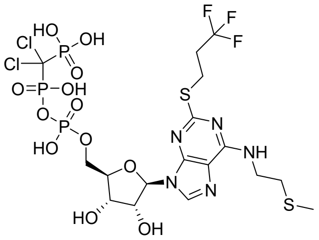	The Medicines Company	776.36
Entresto	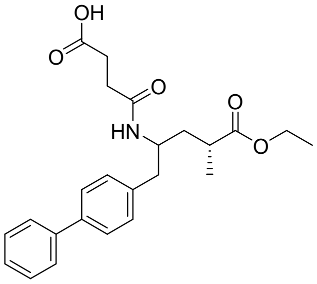	Novartis	411.49
Veltassa	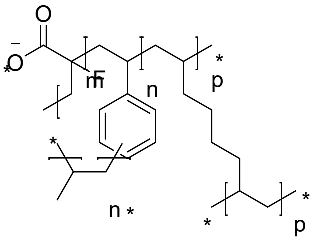	Relypsa	N/A

Uptravi	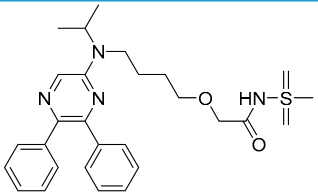	NIPPONSHINYA KU CO. LTD	496.62
Rexulti	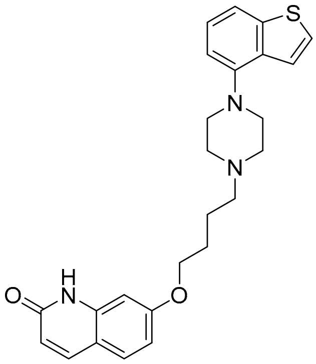	Otsuka	433.57
Vraylar	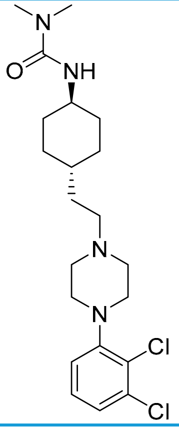	Allergan & Gedeon Richter plc.	427.41
Aristada	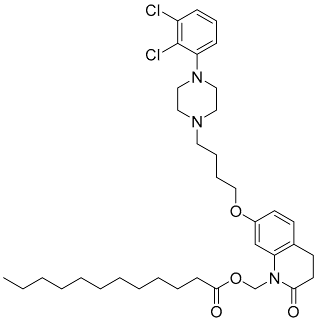	Alkermes	660.71
Daklinza	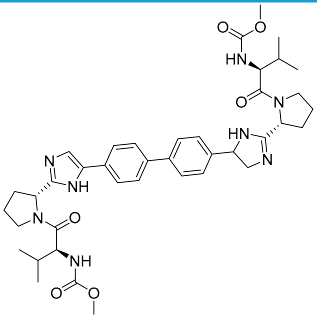	Bristol Myers Squibb	738.88

Genvoya	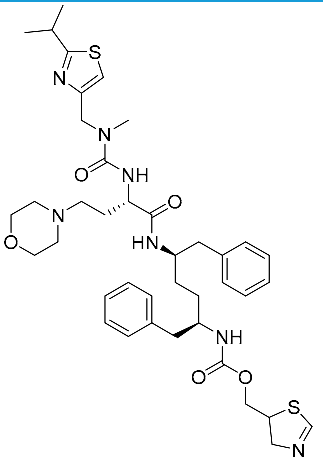 Cobicistat	Gilead Sciences	776.02
	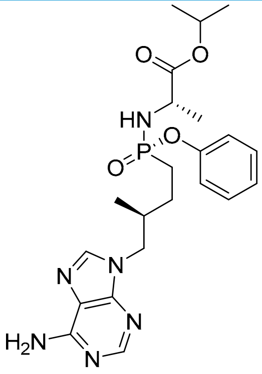 Cobicistat		592.54
	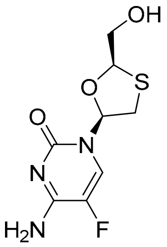 Emtricitabine		247.25
	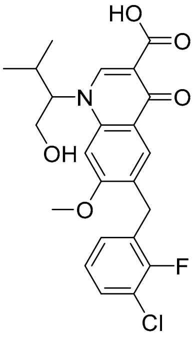 Elvitegravir		447.88
Avycaz	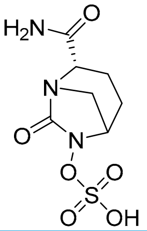	Allergan & Astraeneca	287.23

Cresemba	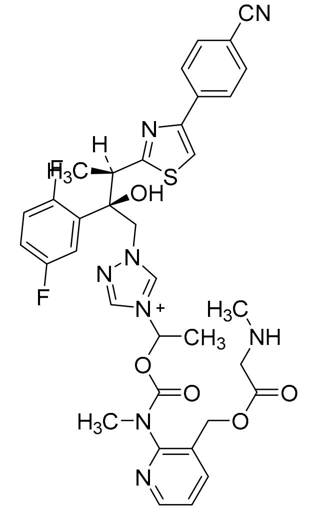	Basilea Pharmaceutica & Astellas Pharmam	814.84
Cholbam	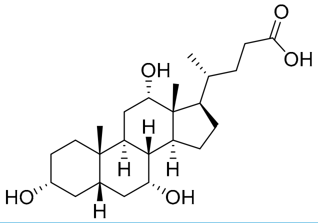	Asklepion pharmaceuticals	408.57
Kybella	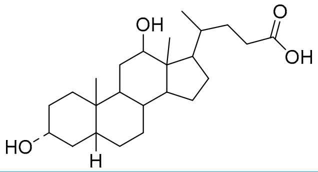	Kythera	392.57
Varubi	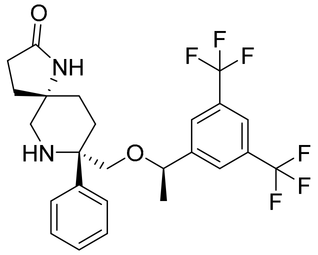	Tesaro	500.48
Xuriden	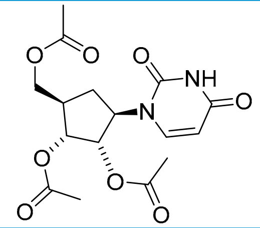	Wellstat Therapeutics	370.31
Orkambi	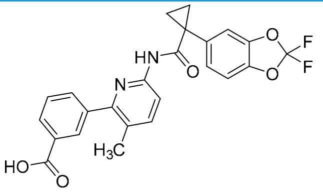	Vertex Pharmaceuticals	452.41
Zurampic	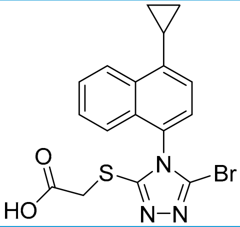	Ardea Biosciences	404.28

Addyi	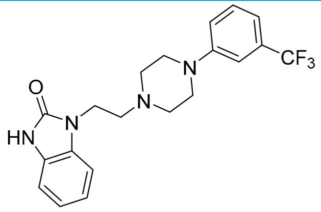	Sprout Pharmaceuticals	390.40
Bridion	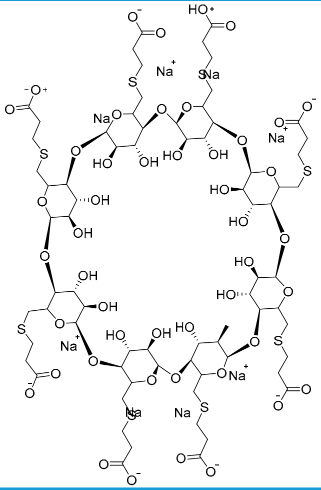	Organon	2022.15
Viberz	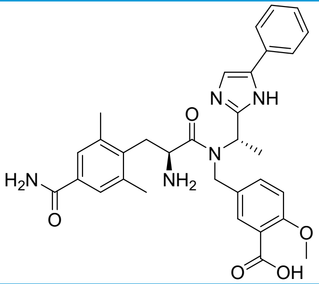	Furiex	569.66

**Drug name 2016**	**Structural formula**	**R&D institutions**	**Molecular Weight (Da)**
Zepatier	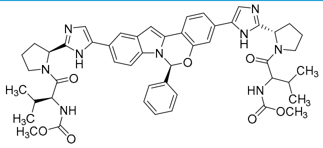 Elbasvir	Merck KGaA	882.02
	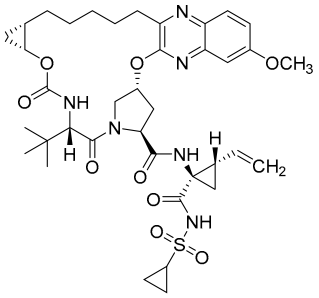 Grazoprevir		766.90

Epclusa	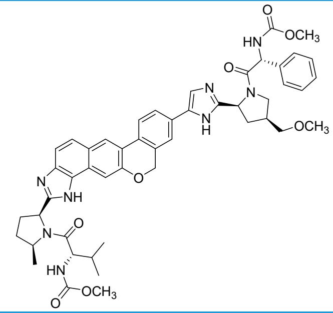	Gilead Sciences	883.00
Briviact	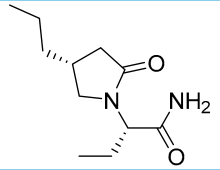	UCB	703.83
Nuplazid	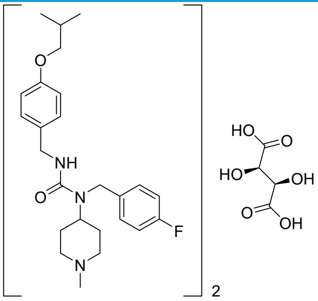	Sdacadia	427.55
Eucrisa	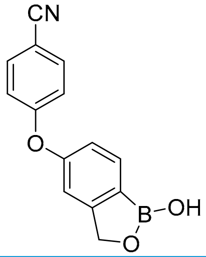	Anacor Pharmaceuticals	251.05
Rubraca	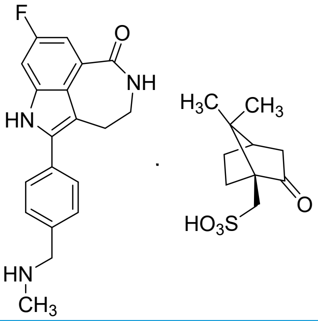	Cloviscancer	323.36

Venclexta	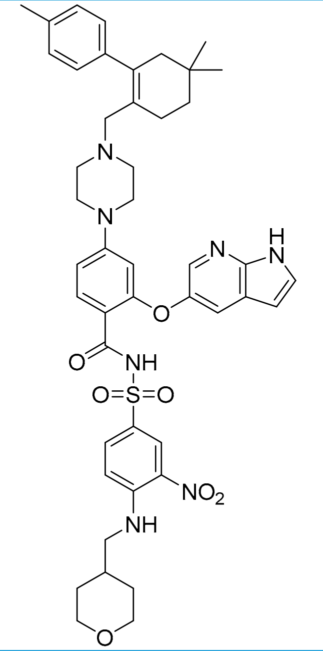	AbbVie & Genentech	868.44
Ocaliva	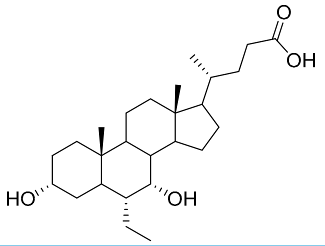	Intercept pharmaceuticals	420.63
Xiidra	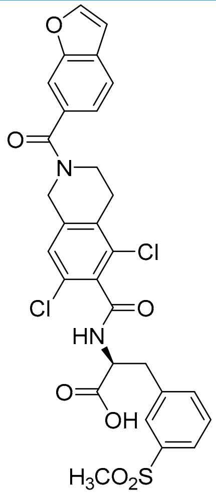	Shire	615.48
**Drug name 2017**	**Structural formula**	**R&D institutions**	**Molecular Weight (Da)**
Baxdela	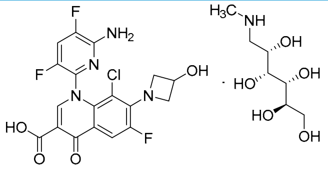	Melinta Therps Inc	440.76

Vabomere	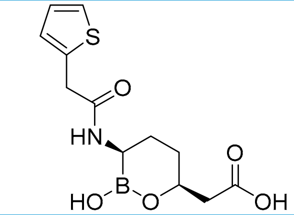	Rempex Pharmaceuticals	297.14
Solosec	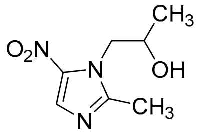	Lupin Pharm	185.18
Xepi	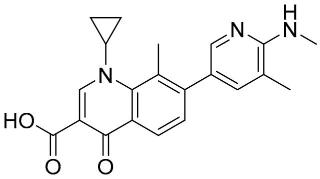	Ferrer Internaional SA	363.41
Vosevi	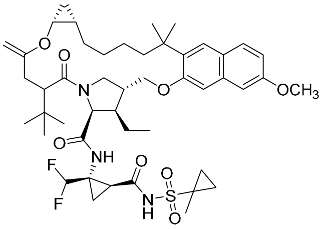	Gilead Sciences	868.93
Mavyret	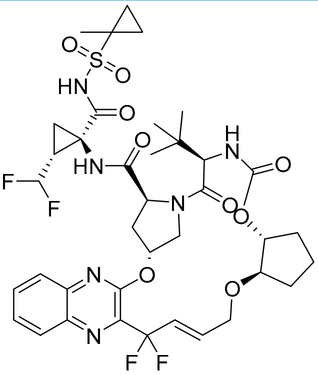 Glecaprevir	Abbvie	838.87
	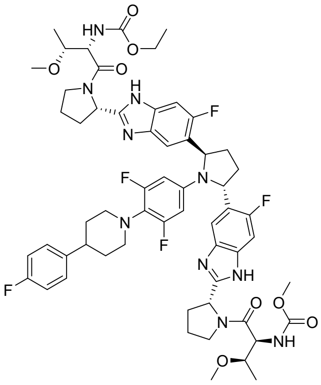 Pibrentasvir		1441.48
Prevymis	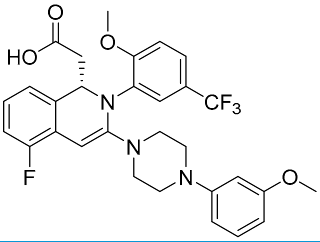	Merck Sharp Dohme	572.55

Kisqal	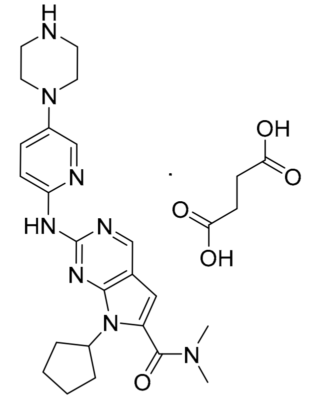	Novartis	434.54
Zejula	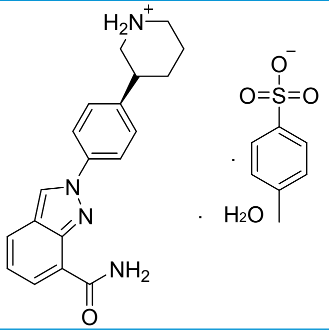	Tesaro Inc	320.39
Alunbrig	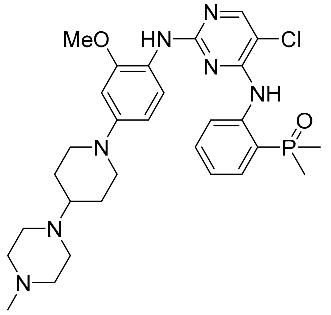	Ariad Pharmaceuticals	584.09
Rydapt	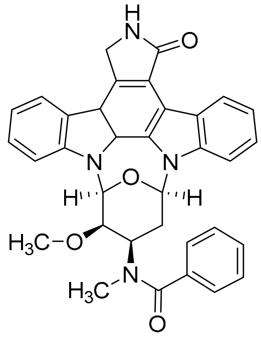	Novartis	570.64
Nerlynx	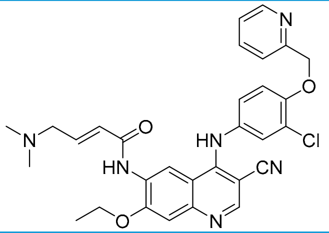	Puma Biotechnology	557.04

Idhifa	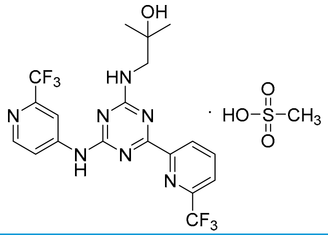	Celgene	473.38
Aliqopa	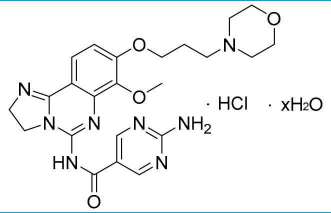	Bayer	480.52
Verzenio	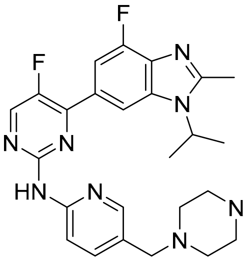	Eli Lilly and Co	506.59
Calquence	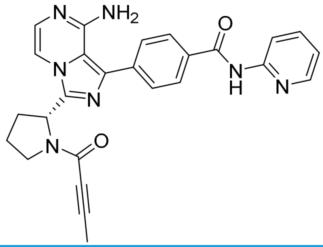	Acerta Pharmaceuticals	465.51
Bevyxxa	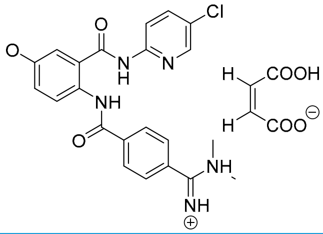	Portola Pharmaceuticals	451.91
Xadago	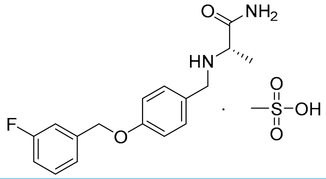	US Worldmeds LLC	302.34
Benznidazole	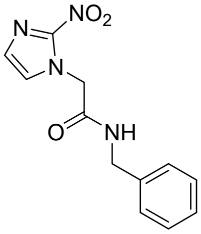	Chemo Reserch SL	260.25
Steglatro	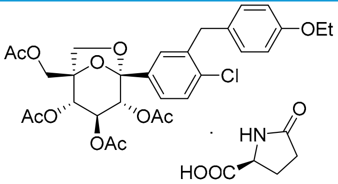	Merck Sharp & Dohome Crop	436.88

Xermelo	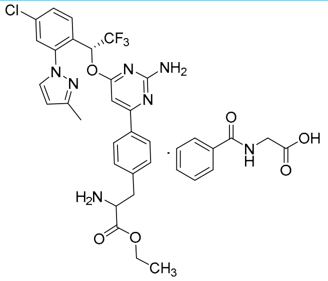	Lexicon Pharmaceutical	574.98
Emflaza	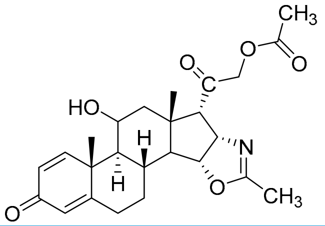	PTC Therap	441.52
Symproic	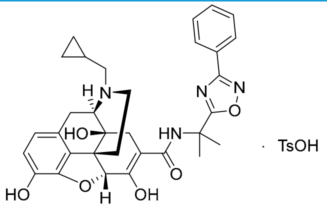	SHIONOGI & CO., LTD	570.64
Austedo	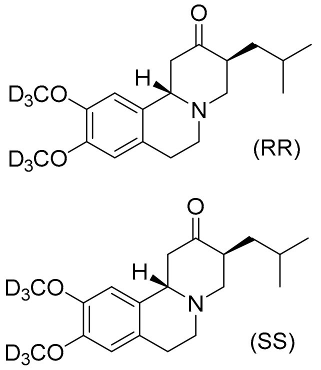	Teva Branded Pharmaceuticals	323.47
Ingrezza	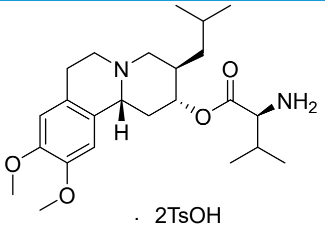	Neurocrine Biosciences Inc	418.57
Radicava	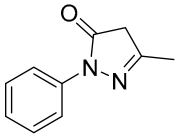	Mitsubishi Tanabe	174.20
Vyzulta	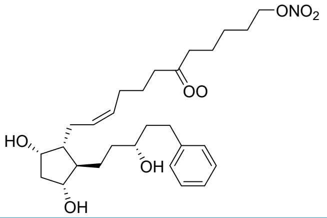	Bausch and Lomb Inc	507.62

Rhopressa	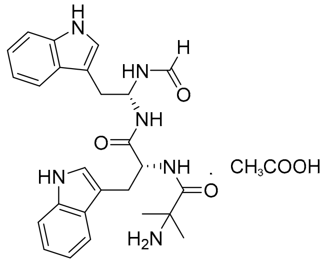	Aerie Pharmaceuticals	453.54
Macrilen	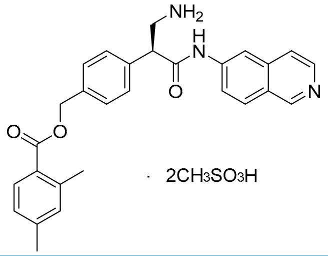	Aeterna Zentaris GmbH	474.55

**Drug name 2018**	**Structural formula**	**R&D institutions**	**Molecular Weight (Da)**
Biktarvy	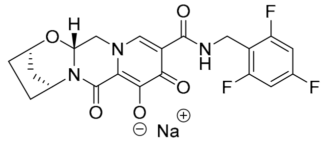 Bictegravir	Gilead Science	449.38
	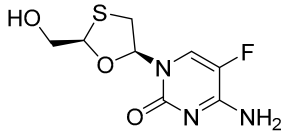 Emtricitabine		247.25
	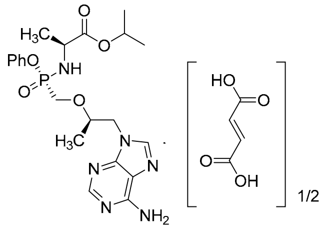 TAF		476.47
Symdeko	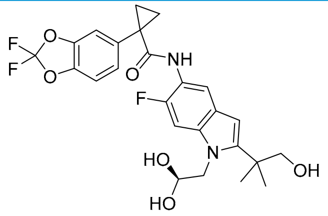 Tezacaftor	Vertex Pharmaceuticals	520.50
	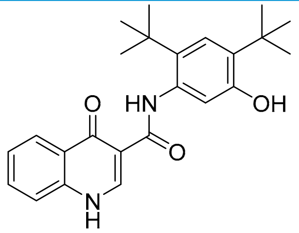 Ivacaftor		392.49

Erleada	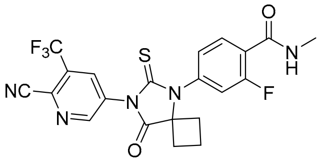	University of California	477.43
Tavalisse	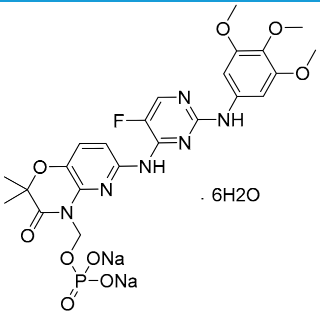	Rigel Pharma	580.46
Akynzeo	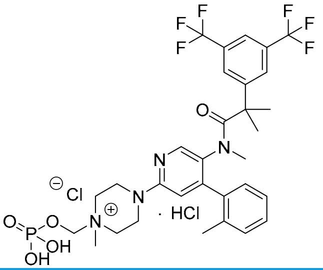	Helsinn Hlthcare	688.60
Lucemyra	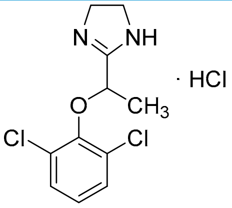	US Worldmeds LLC	295.59
Doptelet	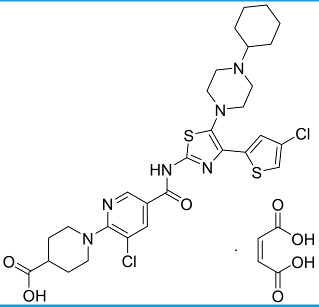	Akarx	649.65
Olumiant	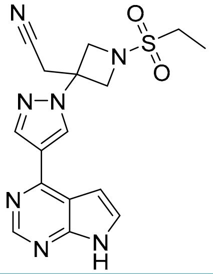	Eli Lilly Company	371.42
Moxidectin	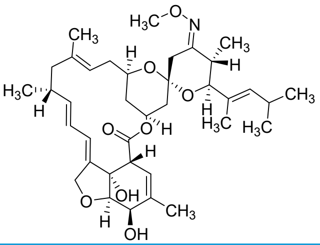	Novartis & Daiichi Sankyo	639.82

Epidiolex	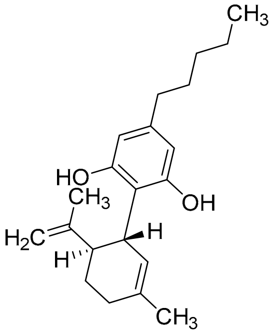	Gw Research	314.47
Zemdri	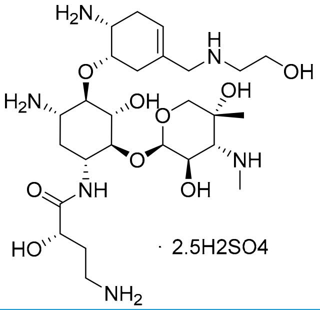	Achaogen	590.72
Braftiovi	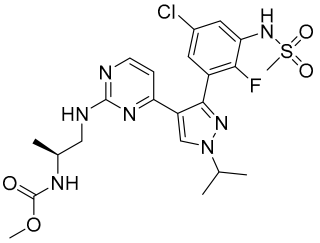	Array Biopharma	540.01
Mektovi	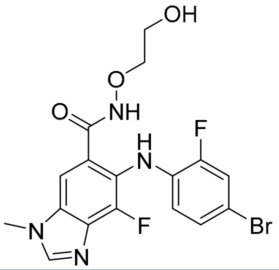	Array Biopharma（small biotech）	441.23
TPOXX	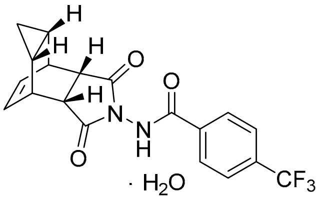	Siga Technologies	380.35
Tibsovo	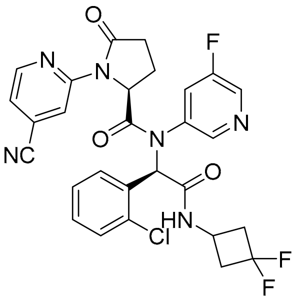	Agios Pharms	582.96

Krintafel	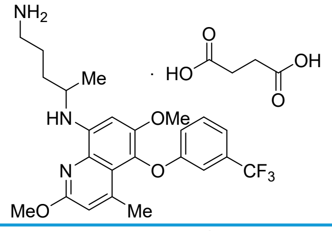	GSK Plc	463.49
Orilissa	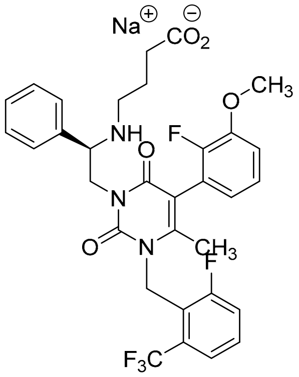	ABBV	653.57
Mulpleta	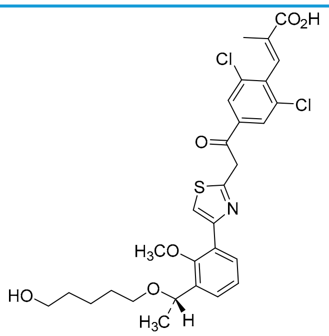	SHIONOGI & CO., LTD.	591.55
Annovera	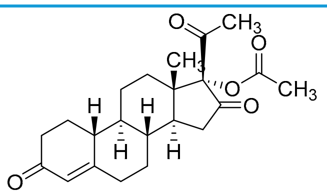	Therapeuticsmd	372.46
Galafold	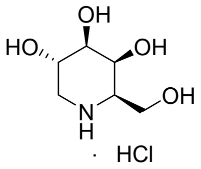	Therapeuticsmd	163.17
Diacomit	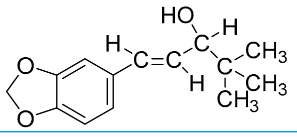	Biocodex Sa	234.29

Xerava	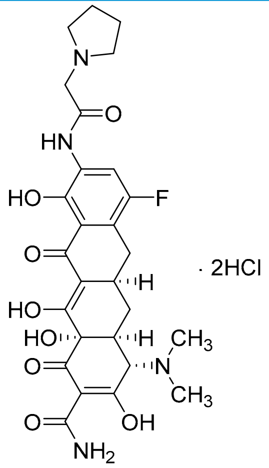	Tetraphase Pharms	558.56
Pifeltro	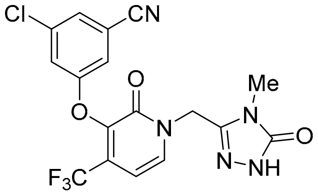	Merck Sharp & Dohme	425.75
Copikra	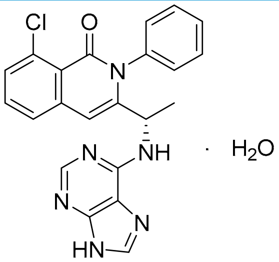	Verastem	416.86
Vizimpro	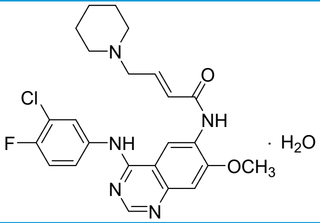	Pfizer	469.94
Seysara	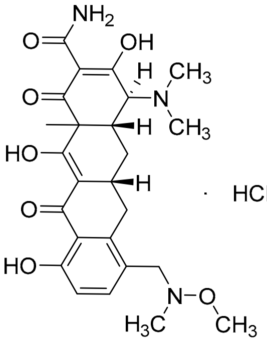	Allergan Aesthetics	532.96

Nuzyra	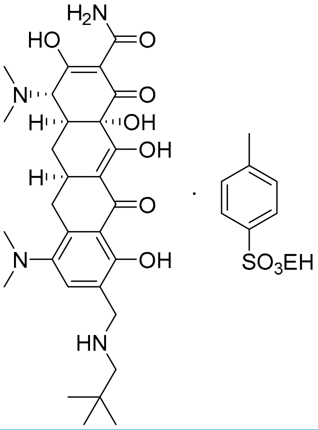	Genentech	556.65
Talzenna	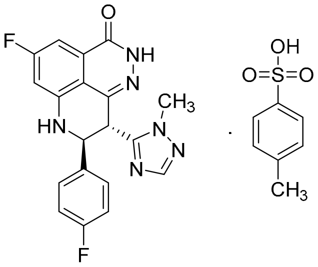	Pfizer	380.35
Xofluza	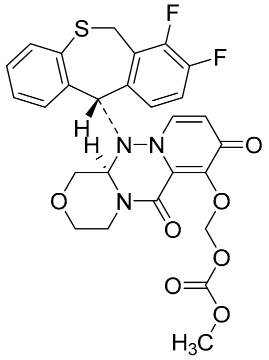	Genentech	571.55
Lorbrena	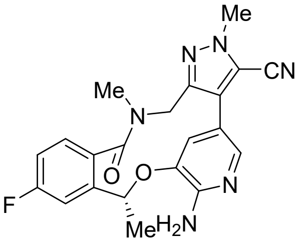	Pfizer	406.41
Yupelri	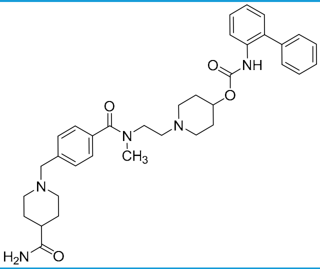	Theravance Biopharma	597.75

DAemcolo	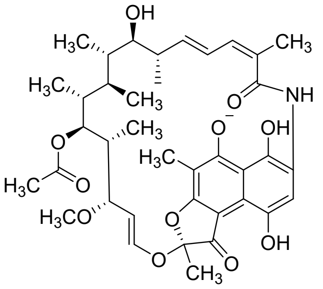	Astellas Pharma Inc	695.75
Daurisom	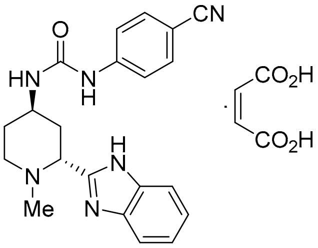	Pfizer	374.44
Vitrakvi	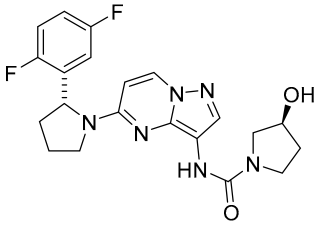	Loxo cancer	428.44
Firdapse	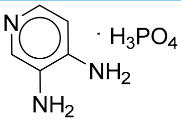	Catalyst Pharma	109.13
Xospata	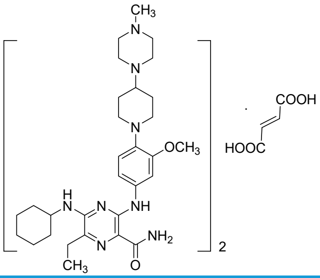	Astellas Pharma Inc	552.71
Motegrity	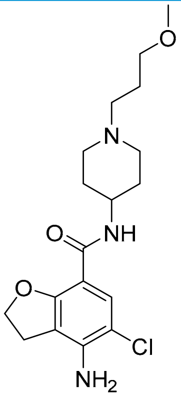	Shire	367.87
**Drug name 2019**	**Structural formula**	**R&D institutions**	**Molecular Weight (Da)**

Piqray	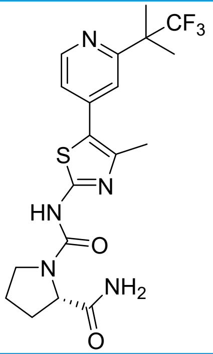	Novartis	441.47
Vyleesi	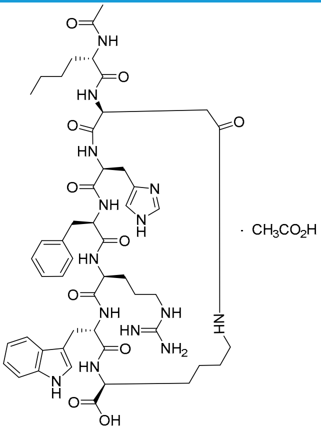	AMAG Pharmaceuticals and Palatin Technologies	1025.16
Zulresso	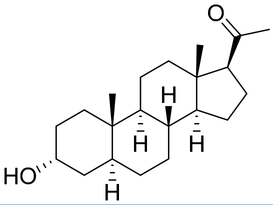	Sage Therapeutics	318.50
Rozlytrek	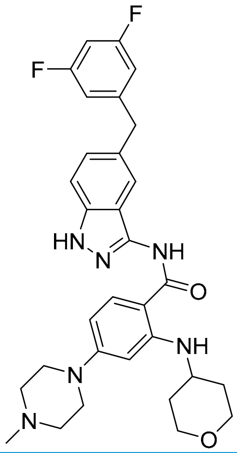	Lgnyta	560.64
Balversa	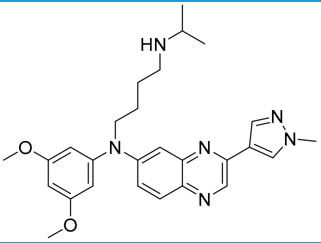	Genentech, Inc	446.54

Inrebic	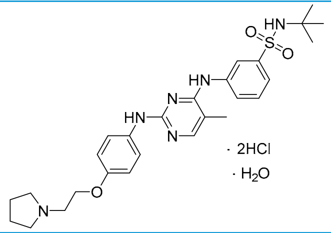	Celgene	524.68
Xenleta	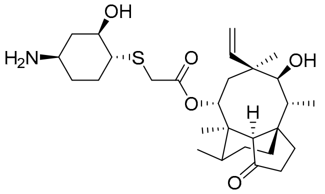	Nabriva Therapeutics	507.73
Turalio	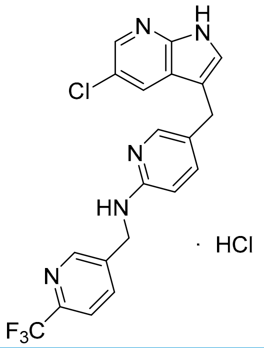	Daiichi Sankyo	417.81
Pretomanid	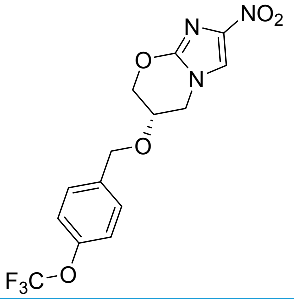	TB Alliance	359.26
Seysara	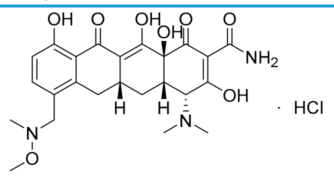	Almiral	523.96
Xpovio	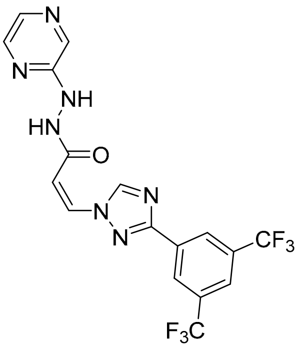	Karyopharm Therapeutics	443.31
Mayzent	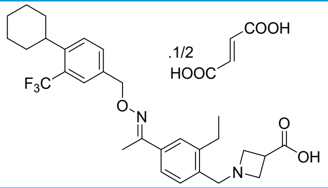	Novartis	516.60

Sunosi	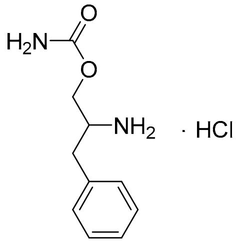	Jazz	194.23
Aklief	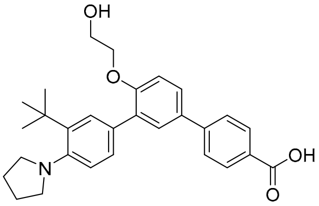	Galderma	459.58
Rinvoq	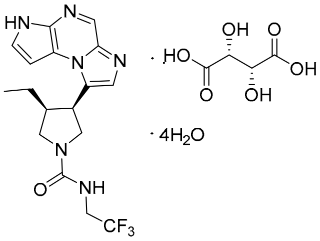	AbbVie	380.37
Oxbryta	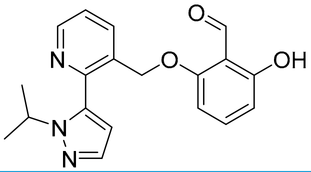	GBT	337.37
Brukinsa	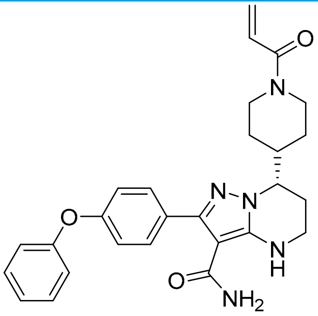	Beigene, Ltd	471.55

**Drug name 2020**	**Structural formula**	**R&D institutions**	**Molecular Weight (Da)**
Ayvakit	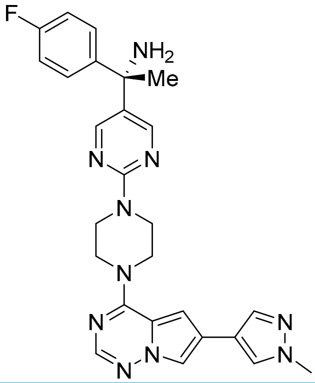	Blueprint Medicines	498.56

Tazverik	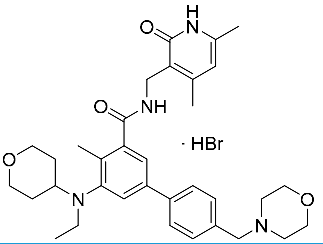	Epizyme	572.75
Nexletol	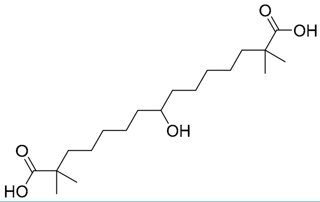	Esperion Therapeutics	344.49
Barhemsys	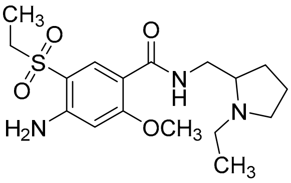	Acacia	369.48
Nurtec odt	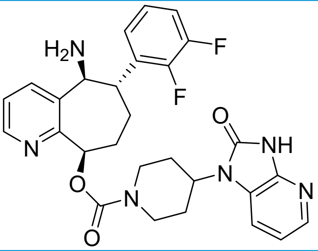	Biohaven	534.56
Isturisa	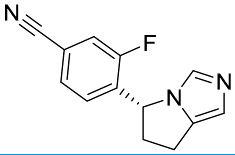	Recordati	227.24
Zeposia	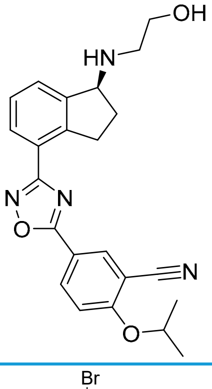	Bristol-Myer Squibb	404.46
Koselugo	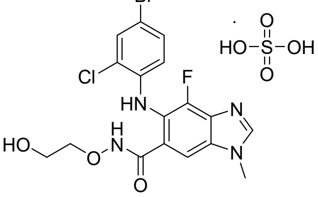	AstraZeneca	457.68
Tukysa	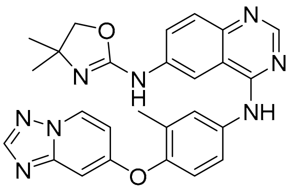	Seattle Genetics	480.52

Pemazyre	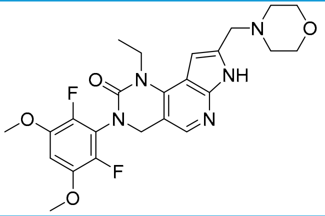	Incyte Corporation	487.50
Ongentys	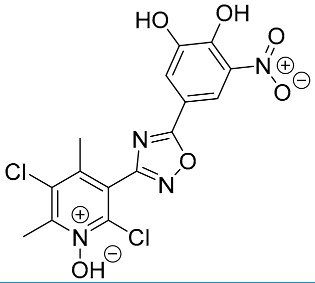	Neurocrine Biosciences	413.17
Tabrecta	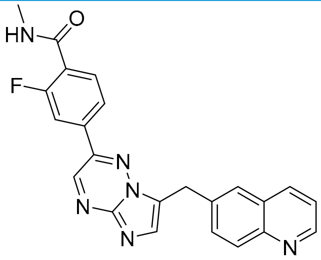	Novartis	412.42
Retevmo	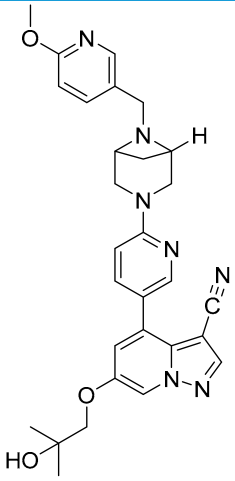	Eli Lilly	525.60
Qinlock	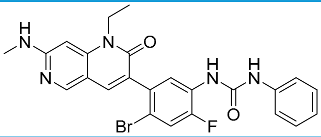	Deciphera Pharmaceuticals	510.36
Cerianna	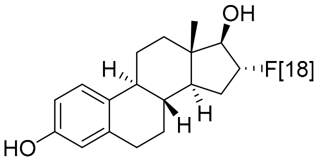	Zionexa (small pharm)	271.38
Artesunate	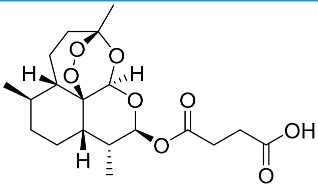	Amivas	384.42
Tauvid	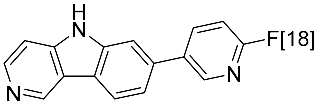	Eli Lilly	244.48

Zepzelca	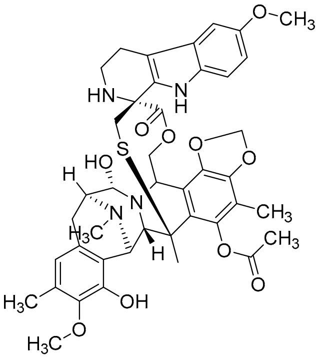	Jazz Pharmaceuticals& PharmaMar	784.87
Dojolvi	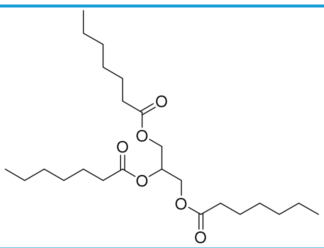	Uitragenyx Pharmaceutical	428.61
Rukobia	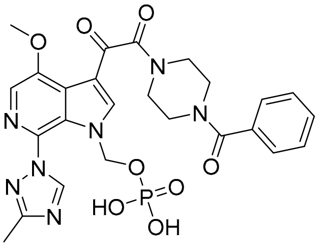	ViiV Healthcare	583.49
Byfavo	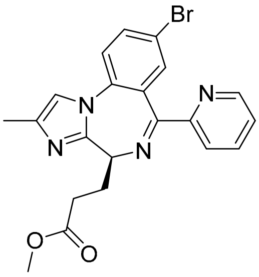	Acacia Pharma	439.31
Inqovi	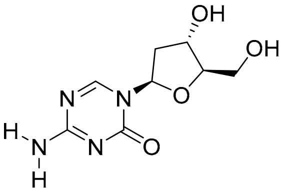 cedazuridine	Otsuka Pharma	228.21
Xeglyze	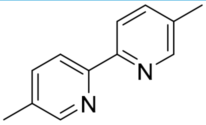	Dr.Reddy	184.24
Lampit	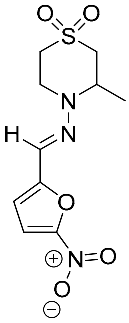	Bayer Health Care	287.30

Olinvyk	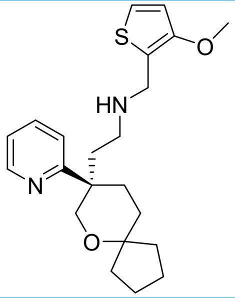	Trevena	386.55
Evrysdil	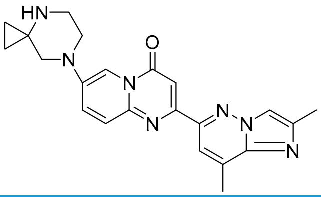	Roche	401.46
Winlevi	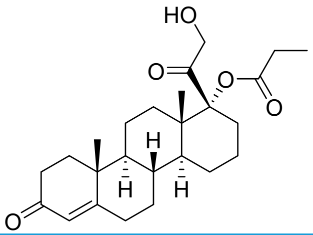	Cassiopea	402.52
Gavreto	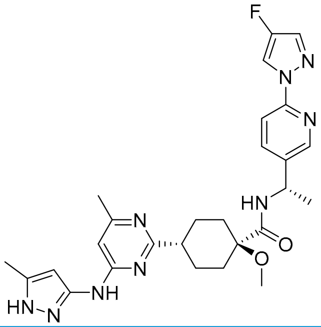	Blueprint Medicines	533.60
Veklury	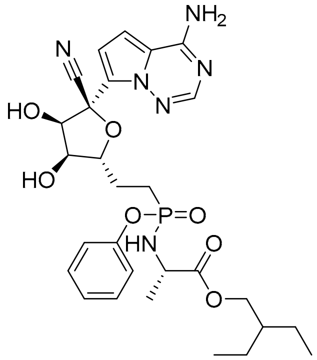	Gilead Sciences	602.58
Zokinvy	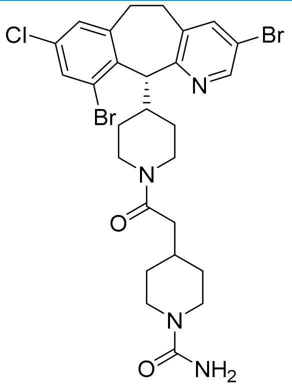	Eiger	638.82

Imcivree	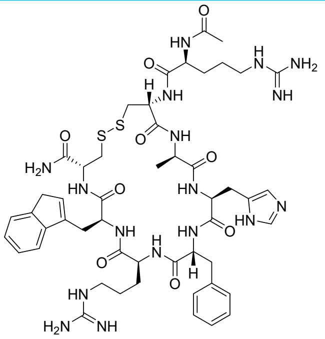	Rhythm Pharmaceuticals Inc	1116.33
Psma-11	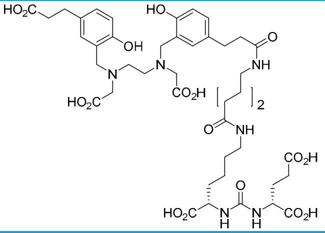	University of California	946.99
Orla-deyo	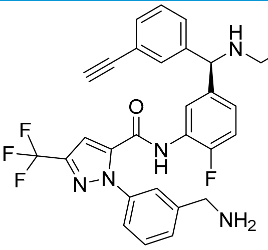	BioCryst	562.56
Klisyri	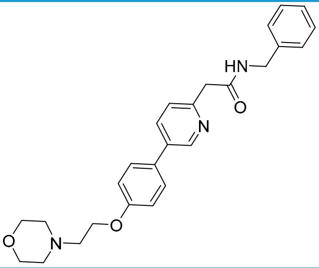	Athenex Inc	431.53
Orgovyx	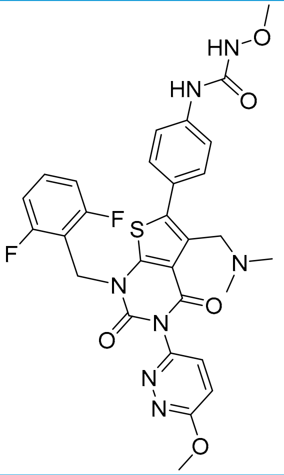	Myovant Sciences	623.63
Gemtesa	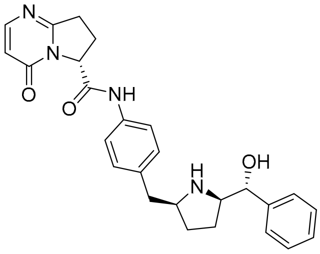	Urovant Sciences	444.53

<table>
<thead>
<tr><th>Drug name
2021</th><th>Structural formula</th><th>R&D
institutions</th><th>Molecular Weight (Da)</th></tr>
</thead>
<tbody>
<tr><td>Vericiguat</td><td>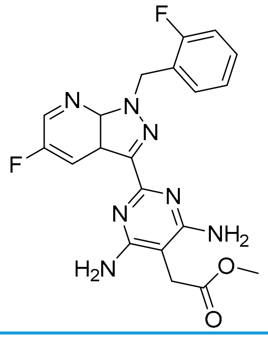</td><td>Bayer & MSD</td><td>426.38</td></tr>
<tr><td rowspan="2">Cabenuva</td><td>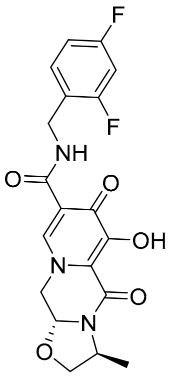
Cabotegravir</td><td rowspan="2">ViiV
Healthcare</td><td>405.35</td></tr>
<tr><td>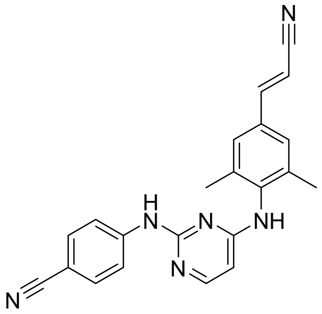
Rilpivirine</td><td>366.42</td></tr>
<tr><td>Tepotinib</td><td>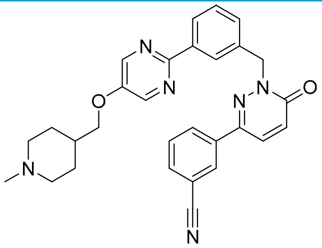</td><td>Merck</td><td>492.58</td></tr>
</tbody>
</table>

Umbralisib	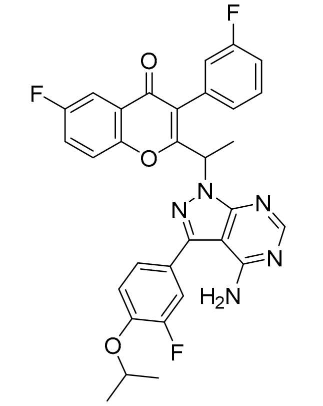	TG Therapeutics	571.55
Trilacicilib	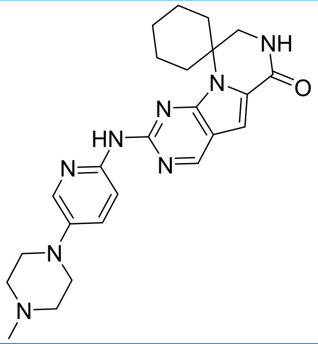	G1 Therapeutics	446.55
Fosdenopterin,	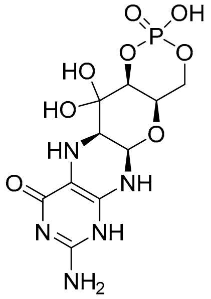	Origin Biosciences	363.22
Azstarys	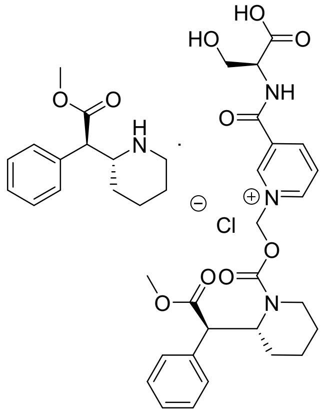 Serdexmethylphenidate	KemPharm	499.50
	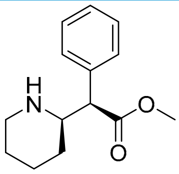 Dexmethylphenidate		233.31

Tivozanib	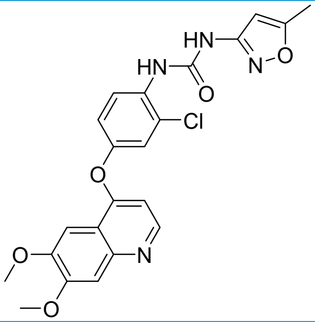	Aveo Pharmaceuticals	446.55
Ponesimod	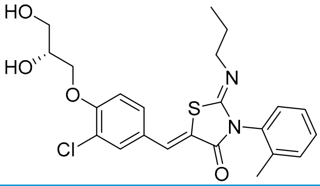	Johnson	363.22
Viloxazine	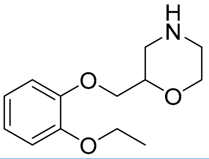	Supernus Pharms	499.50
Drospirenone	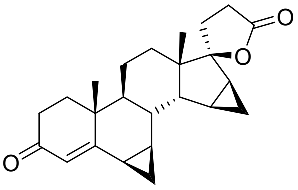	Mayne Pharma	233.31
Sotorasib	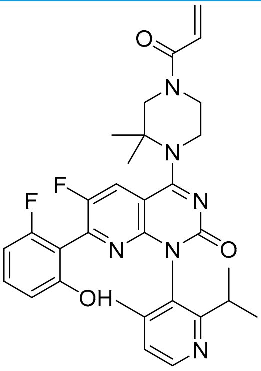	Amgen	560.59
Infigratinib	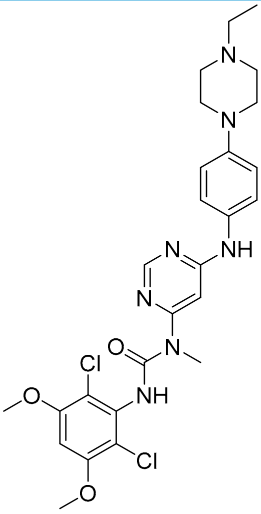	QED Therapeutics	560.48

Samidorphan	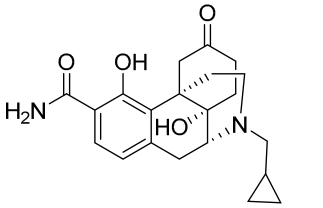	Alkermes	370.40
Ibrexafungerp	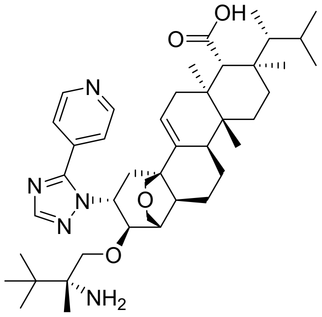	SCYNEXIS	730.00
Finerenone	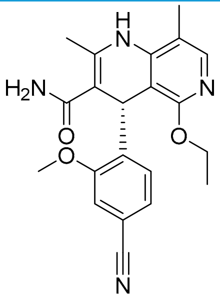	Bayer HealthCare Pharmaceuticals	378.42
Fexinidazole	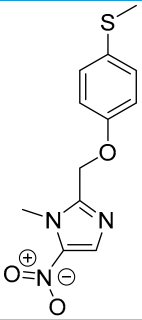	Sanofi-Aventis	279.31
Belumosudil	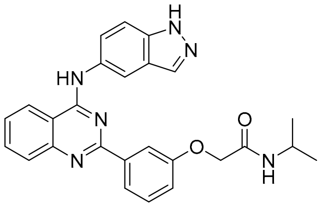	Kadmon Pharmaceuticals	452.51
Odevixibat	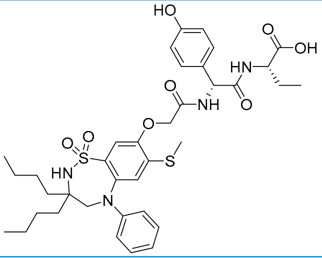	Albireo Pharma	740.93

Belzutifan	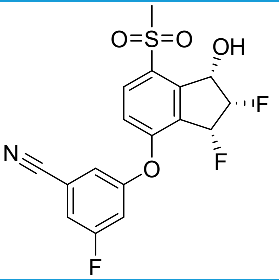	Merck Sharp & Dohme	383.34
Difelikefalin	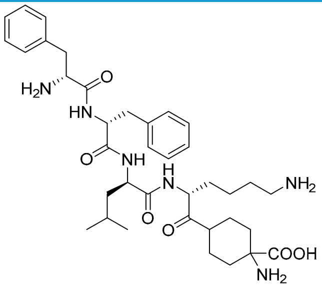	Cara Therapeutic	679.85
Mobocertinib	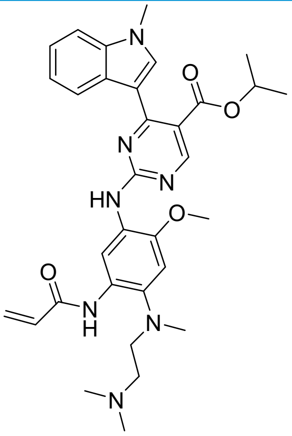	Merck Sharp & Dohme	585.70
Atogepant	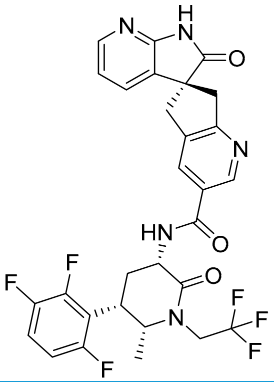	AbbVie	603.52
Maralixibat	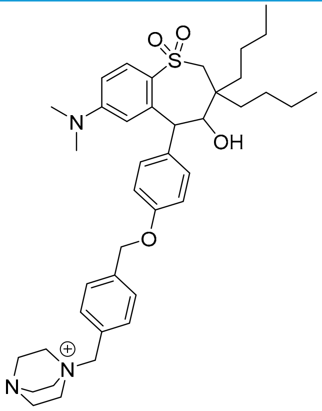	Mirum Pharma	710.41

**Drug name 2022**	**Structural formula**	**R&D institutions**	**Molecular Weight (Da)**
Quviviq	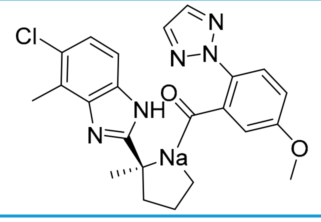	Idorsia	459.91
Cibinqo	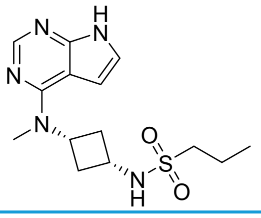	Pfizer	323.42
Pyrukynd	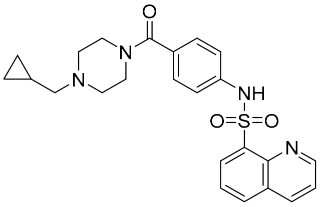	Agios	450.56
Vonjo	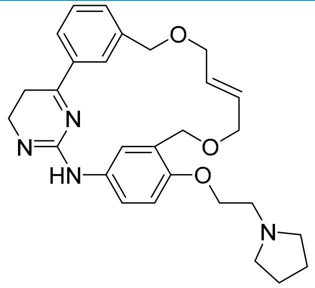	CTI BioPharma	474.61
Ztalmy	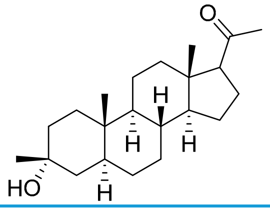	Marinus	332.54
Vivjoa	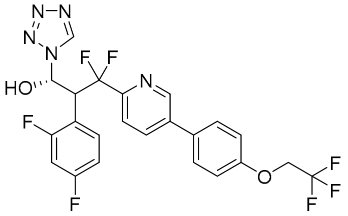	Mycovia Pharma	527.40
Camzyos	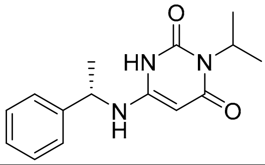	BMS	273.34

Voquezna	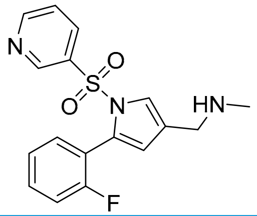	Phathom Pharma	345.39
Vtama	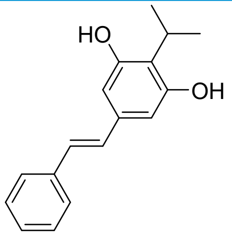	Dermavant Sciences	254.33
Sotyktu	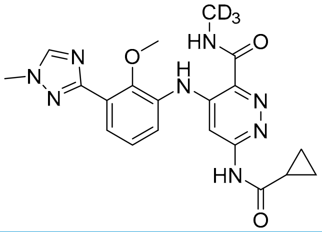	BMS	425.47
Omlonti	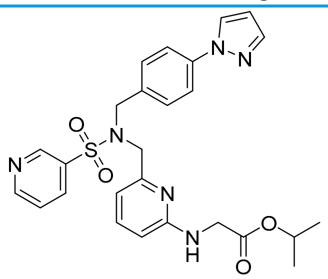	Santen & UBE	520.61
Relyvrio	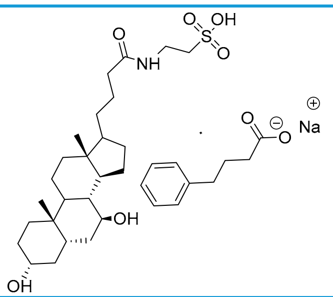	Amylyx	458.68
Lytgobi	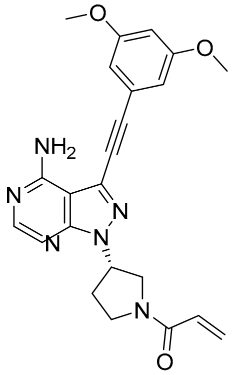	Taiho cancer	431.48
Rezlidhia	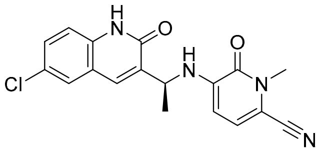	Rigel Pharma	354.79

Krazati	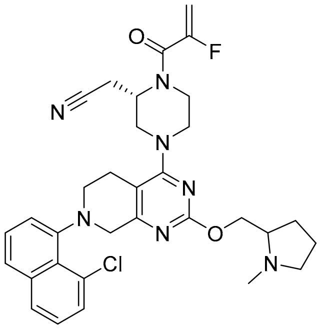	Mirati	604.13
Sunlenca	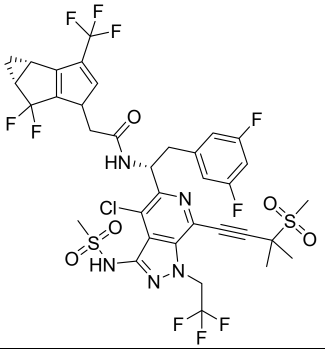	Gilead	890.21

Supplementary Table S2. The Physicochemical properties, Indication, and Route of administration of small molecule drugs approved by the FDA

Drug Name (Trade Name, Original Name) 2012	Indications	ClogP	Route Of Administration
1. Orkambi, ivacaftor [b]	Cystic Fibrosis	3.82	Oral
2. Xeljanz, tofacitinib [b]	RA	1.55	Oral
3. Xtandi, enzalutami [b]	CRPC	3.35	Oral
4. Eliquis, apixaban [b]	VTE	1.89	Oral
5. Picato, ingenol mebutate [a]	PKC	3.41	Topical
6. Inlyta, axitinib [b]	Advanced Renal Carcinoma	3.33	Oral
7. Erivedge, vismodegib [b]	Basal Cell Carcinoma Of	2.74	Oral

	Skin		
8. Zioptan, tafluprost [b]	Open Angle Glaucoma	4.26	Topical
9. Stendra, avanafil [b]	ED Osteoporosis	2.36	Oral
10. Belviq, lorcaserin [b]	BMI≥27 Obesity	3.24	Oral
11. Mybetriq, mirabegron [a]	OAB	1.32	Oral
12. Kyprolis, carfilzomib [b]	Multiple Myeloma (MM)	6.40	Parenteral
13. Tudorza pressair, aclidinum [b]	Chronic Obstructive Pulmonary Disease (COPD)	2.34	Inhalant
14. Bosulif, bosutinib [b]	Chronic Myelogenous Leukemia (CML)	4.93	Parenteral
15. Aubagio, teriflunomid [b]	Multiple Sclerosis (MS)	2.13	Oral
16. Stivaga, regorafenib [b]	Metastatic Colorectal Cancer	5.19	Oral
17. Fycompa, perampanel [a]	Epilepsy	4.17	Oral
18. Cometriq, cabozantinib [b]	Hepatocellular Carcinoma	4.94	Oral
19. Lclusig, ponatinib [b]	Chronic	5.77	Oral

	Myelogenous Leukemia (CML)		
20. Juxtapid, lomitapide [a]	Homozygous Familial	7.00	Oral
21. Sirturo, bedaquiline [a]	Tuberculosis	7.85	Oral
**Drug Name (Trade Name, Original Name) 2013**	**Indications**	**ClogP**	**Route Of Administration**
1. Pomalyst, pomalidomide [b]	Multiple Myeloma	-0.19	Oral
2. Tafinlar, dabrafenib mesylate [b]	Melanoma	4.61	Oral
3. Mekinist, trametinib dimethyl sulfoxide [b]	Melanoma Cancer	4.84	Oral
4. Gilotrif, afatinib dimaleate [b]	Mtastatic Non-Small Cell Lung Cancer	4.34	Oral
5. Imbruvica, ibrutinib [a]	MCL	4.07	Oral
6. Tivicay, dolutegravir sodium [b]	HIV	-0.73	Oral
7. Olysio, simeprevir [b]	HCV	5.52	Oral
8. Sovaldi, sofosbuvir [a]	HCV	0.84	Oral
9. Nesina, alogliptin benzoate [b]	NIDDM	0.99	Oral
10. Invokana, canagliflozin [a]	NIDDM	4.55	Oral
11. Adempas, riociguat [a]	PAH	2.05	Oral
12. Opsumit, macitentan [b]	PAH	4.30	Oral
13. Breo ellipta, vilanterol trifenatate [b]	COPD	3.19	Inhalant
14. Bromide, umeclidinium [b]	COPD	1.02	Oral
15. Osphena, ospemifene [b]	Female	5.56	Oral

	Menopausal Vulva		
16. Tecfidera, dimethyl fumarate [a]	MS	0.78	Oral
17. Brintellix, vortioxetine hydrobromide [b]	MDD	4.92	Oral
18. Aotiom, eslicarbazepine acctate [b]	Epilepsy	1.50	Oral
19. Luzu, luliconazole [b]	Fungal Infections	3.49	Topical
**Drug Name (Trade Name, Original Name) 2014**	**Indications**	**ClogP**	**Route Of Administration**
Zykadia, ceritinib [b]	NSCLC	5.43	Oral
Beleodaq, belinostat [b]	PTCL	1.18	Parenteral
Zydelig, idelaisib [a]	CLL	3.62	Oral
Ledipasvir [b]	HCV	6.71	Oral
Rapivab, peramivir [b]	Anti-Influenza	-1.31	Parenteral
Viekira pak[b] ombitasvir	HCV	8.03	Oral
paritaprevir	HCV	6.28	Oral
dasabuvir	HCV	4.53	Oral
Impavido, miltefosine [a]	Kala-Azar	-2.38	Oral
Farxiga, dapagliflozin [b]	DMII	3.37	Oral
Jardiance, empagliflozin [b]	DMII	2.59	Oral
Northera, droxidopa [a]	PD	-2.48	Oral
Hetlioz, tasimelteom [b]	Non-24	1.89	Oral
Belsomra, suvorexant [a]	Insomnia	4.79	Oral
Cerdelga, eliglustat tartrate [b]	Type I Gaucher Disease	4.83	Oral

Movantik, naloxegol oxalate [b]	OIC	1.17	Oral
Striverdi respimat, olodaterol hydrochloride [b]	COPD	1.44	Inhalant
Sivextro, tedizolid phosphate [b]	Bacterial Skin	-0.50	Oral
Xtoro, finafloxacin [b]	Acute External Otitis	0.43	Topical
Zerbaxa, ceftolozane [b]	RTI	-9.85	Parenteral
Dalvance, dalbavancin hydrochloride [b]	ABSSSI	N/A	Parenteral
Jublia, efinaconazole [b]	Onychomycosis	2.15	Topical
Kerydin, tavaborole [a]	Tinea Unguium	1.24	Topical
Otezla, apremilast [a]	Psoriasis	1.46	Oral
Zontivity, vorapaxar sulfate [a]	ACS	4.68	Oral
Akynzeo, netupitant [b]	CINV	6.51	Oral
Esbrie, pirfenidone [b]	IPF	2.40	Oral
Ofev, nintedanib [a]	IPF	3.08	Oral
**Drug Name (Trade Name, Original Name) 2015**	**Indications**	**ClogP**	**Route of administration**
Ibrance, palbociclib [a]	Breast Cancer	2.20	Oral
Lenvima, lenvatinib [b]	DTC	3.35	Oral
Fardak, panobinostat [b]	Multiple Myeloma	2.64	Oral
Odomzo, sonidegib [b]	Basal Cell Carcinoma	5.51	Oral
Lonsurf, trifluridine [b]	Herpes Zoster Keratitis	-0.41	Oral
Cotellic, trifluridine[b]	Metastatic	6.16	Oral

		Melanoma		
Ninlaro, ixazomib [b]		Multiple Myeloma	1.10	Oral
Yonndelis, trabectedin [b]		Liposarcoma	2.90	Parenteral
Tagrisso, osimertinib [b]		Non-Small Cell Lung Cancer	5.01	Oral
Alecensa, alectinib [b]		Non-Small Cell Lung Cancer	5.53	Oral
Corlanor, ivabradine [a]		Heart Failure	2.99	Oral
Savaysa, edoxaban [b]		Thromboembolism	2.27	Parenteral
Kengreal, cangrelor [b]		CAD	1.56	Parenteral
Entresto, sacubitril [a]		NYHAII-IV	4.47	Oral
Veltassa, patiromer [b]		Hyperkalemia	N/A	Oral
Uptravi, selexipag [b]		Pulmonary Arterial Hypertension (PAH)	5.00	Parenteral
Rexulti, brexpiprazole [b]		Adult Schizophrenia	4.65	Oral
Vraylar, cariprazine [b]		Manic	4.98	Oral
Aristada, aripiprazole lauroxil [b]		Schizophrenia	10.80	Parenteral
Daklinza, daclatasvir [b]		HCV	5.64	Oral
Genvoya [b]	cobicistat	HIV	3.59	Oral
	tenofovir alafenamide fumarate	HIV	2.71	Oral
	emtricitabine	HIV	-1.29	Oral
	elvitegravir	HIV	4.56	Oral

Avycaz, avibactam sodium [b]	Intraperitoneal Infections	-1.63	Parenteral
Cresemba, isavuconazoium sulfate [b]	Infections Caused By Mucor	-1.15	Oral/Parenteral
Cholbam, cholic acid [b]	Cholesterol Excretion	2.43	Oral
Kybella, deoxycholic acid [b]	Submaxillary Fat	4.51	Parenteral
Varubi, rolapitant [b]	Chemotherapy	4.71	Oral
Xuriden, uridine triacetate [a]	Hereditary Whey Aciduria	-0.22	Oral
Orkambi, lumacaftor [a]	CF	6.05	Oral
Zurampic, lesinurad [b]	Metabolic Arthritis	4.01	Oral
Addyi, flibanserin [a]	HSDD	4.73	Oral
Bridion, sugammadex [a]	Neuromuscular Block	N/A	Parenteral
Viberzi, eluxadoline [b]	IBS-D	0.72	Oral
**Drug Name (Trade Name, Original Name) 2016**	**Indications**	**ClogP**	**Route of administration**
Zepatier [b] elbasvir	HCV	6.66	Oral
Zepatier [b] grazoprevir	HCV	6.32	Oral
Epclusa, brivaracetam [b]	HCV	5.70	Oral
Briviact, brivaracetam [b]	Localized Epileptic Seizures	1.01	Parenteral
Nuplazid, pimavanserin [b]	Mental	4.11	Oral

	Disorder		
Eucrisa, crisaborole [b]	Atopic Dermatitis	2.63	Topical
Rubraca, rucaparib [b]	Ovarian Cancer	3.01	Oral
Venclexta, venetoclax [a]	CLL	10.10	Oral
Ocaliva, obeticholic acid [a]	PBC	5.36	Oral
Xiidra, benznidazol [a]	DED	2.28	Topical
**Drug Name (Trade Name, Original Name) 2017**	**Indications**	**ClogP**	**Route of administration**
Baxdela, delafloxacin [b]	Fluoroquinolone Antibiotic	1.25	Parenteral
Vabomere, secnidazole [b]	cUTI	0.53	Parenteral
Solosec, secnidazole [b]	Intestinal Amebiasis	-0.15	Oral
Xepi, ozenoxacin [b]	Nonfluorinated Quinolone	3.39	Topical
Vosevi, voxilaprevir [b]	HCV	11.31	Oral
Mavyret [b] glecaprevir	HCV	4.00	Oral
pibrentasvir	HCV	7.73	Oral
Prevymis, letermovir [a]	CMV	7.31	Oral/Parenteral
Kisqal, ribociclib [b]	MBC	1.80	Oral
Zejula, niraparib [b]	ROC	3.18	Oral
Alunbrig, midostaurin [b]	Lung Cancer	1.72	Oral
Rydapt, midostaurin [a]	AML	2.50	Oral
Nerlynx, enasidenib [b]	BC	5.00	Oral
Idhifa, enasidenib [a]	AML	3.76	Oral
Aliqopa, copanlisib [b]	R/R FL	1.11	Parenteral
Verzenio, abemaciclib [b]	Breast Cancer	4.77	Oral

Calquence, acalabrutinib [b]		Ovarian Cancer	1.58	Oral
Bevyxxa, safinamide [b]		CLL	3.61	Oral
Xadago, safinamide [b]		PD	2.49	Oral
Benznidazole [b]		Chagas	0.90	Oral
Steglatro, ertugliflozin [b]		T2DM	5.33	Oral
Xermelo, telotristat ethyl [a]		Carcinoid Syndrome	5.09	Oral
Emflaza, deflazacort [a]		DMD	1.76	Oral
Symproic, naldemedine [b]		OIC	2.82	Oral
Austedo, deutetrabenazine [b]		Huntington's Disease	3.47	Oral
Ingrezza, valbenazine [b]		TD	3.97	Oral
Radicava, edaravone [a]		ALS	1.33	Parenteral
Vyzulta, latanoprostene bunod [b]		IOP<br>OHT	0.78	Topical
Rhopressa, netarsudil [a]		Open-Angle Glaucoma<br>Ocular Hypertension	1.14	Topical
Macrilen, macimorelin acetate [a]		CACS<br>AGHD	5.01	Oral
**Drug Name (Trade Name, Original Name) 2018**		**Indications**	**ClogP**	**Route of administration**
Biktarvy [a]	bictegravir	HIV-1	-3.27	Oral
	emtricitabine	HIV	-1.29	Oral
	TAF	HBV	2.18	Oral
Symdeko [a]	tezacaftor	CF	3.27	Oral
	ivacaftor	CF	3.82	Oral

Erleada, apalutamide [a]	nmCRPC	2.14	Oral
Tavalisse, fostamatinib [a]	ITP	2.22	Oral
Cakynzeo, fosnetupitant [b]	CINV	2.40	Parenteral
Lucemyra, lofexidine [a]	Opioid	3.46	Oral
Doptelet, avatrombopag [a]	ITP<br>CLD	6.41	Oral
Olumiant, baritinib [a]	AA	0.41	Oral
Moxidectin [a]	Heartworm<br>Roundworm	7.18	Oral
Epidiolex, cannabidiol [a]	LGS	6.41	Oral
Zemdri, plazomici [a]	UTI	-2.68	Parenteral
Braftiovi, encorafenib [b]	Melanoma	2.81	Parenteral
Mektovi, tecovirimat [a]	Melanoma	3.16	Oral
Tpoxx, tecovirimat [a]	Smallpox	2.94	Oral
Tibsovo, ivosidenib [a]	AML	1.81	Oral
Krintafel, tafenoquine [a]	Antimalarial Drugs	6.52	Oral
Orilissa, elagolix sodium [a]	Endometriosis	3.24	Oral
Mulpleta, lusutrombopag [a]	Chronic Liver Disease	5.28	Oral
Annovera, segesterone [a]	Female Contraception	2.05	Topical
Galafold, migalastat [a]	Fabry's Disease	-1.42	Oral
Diacomit, stiripentol [a]	Anticonvulsant s	3.21	Oral
Xerava, eravacycline [a]	Intraperitoneal Infections	0.17	Parenteral
Pifeltro, doravirine [a]	HIV-1	2.37	Oral
Copikra, duvelisib [b]	CLL	4.39	Parenteral

	SLL<br>FL		
Vizimpro, dacomitinib [a]	NSCLC	5.75	Oral
Seysara, sarecycline [b]	Acne	2.01	Oral
Nuzyra, omadacycline [a]	Pneumonia	1.38	Oral
Talzenna, talazoparib [a]	Breast Cancer	0.35	Oral
Xofluza, baloxavir marboxil [a]	Influenza	2.36	Oral
Lorbrena, lorlatinib [a]	NSCLC	1.87	Oral
Yupelri, revefenacin [a]	COPD	3.21	Topical
Daemcolo, rifamycin [b]	Leprosy	2.17	Oral
Daurisom, glasdegib [b]	Leukemia	2.75	Oral
Vitrakvi, larotrectinib [a]	Solid Tumors	2.00	Oral
Firdapse, amifampridine [a]	Lambert-Eat-O<br>n Myasthenia<br>Syndrome	0.39	Oral
Xospata, gilteritinib [a]	AML	4.52	Oral
Motegrity, prucalopride, [a]	Constipation	1.41	Oral
**Drug Name<br>(Trade Name, Original Name)<br>2019**	**Indications**	**ClogP**	**Route of administration**
Piqray, alpelisib [a]	Breast cancer	2.13	Oral
Vyleesi, brexanolone [b]	Hyposexual<br>Desire Disorder	-1.79	Parenteral
Zulresso, brexanolone [a]	Postpartum<br>Depression	4.51	Parenteral
Rozlytrek, entrectinb [a]	Solid Tumors	5.22	Oral
Balversa, erdafitinib [a]	Bladder Cancer	4.67	Oral
Inrebic, fedratinib [a]	Myelofibrosis	6.25	Parenteral
Xenleta, lefamulin [a]	Pneumonia	4.24	Parenteral

Turalio, pexidartinib [a]	Giant Cell Tumor Of Ganglion Sheath	3.99	Oral
Pretomanid [a]	MTB	2.79	Oral
Seysara, sarecycline hydrochloride [a]	Acne	6.63	Oral
Xpovio,selinexor [a]	Myeloma	2.68	Oral
Mayzent, siponimod [a]	RMS	6.00	Oral
Sunosi, solriamfetol [a]	Obstructive Sleep Apnea	0.63	Oral
Aklief, trifarotene [a]	Topical Treatment Of Acne	6.31	Topical
Rinvoq, upadacitinib [a]	Rheumatoid Arthritis	2.26	Oral
Oxbryta, voxelotor [a]	Sickle Cell Disease	3.24	Oral
Brukinsa, zanubrutinib [a]	Cell Lymphoma	2.84	Oral
**Drug Name (Trade Name, Original Name) 2020**	**Indications**	**ClogP**	**Route of administration**
Ayvakit, avapritinib [b]	Stromal Tumor	1.60	Oral
Tazverik, tazemetostat [a]	Epithelioid Sarcoma	3.99	Oral
Nexletol, bempedoic acid [a]	Dyslipidemia	3.95	Oral
Barhemsys, amisulpride [b]	Schizophrenia	1.80	Parenteral
Nurtec odt, rimegepant [b]	Migraine	3.68	Oral

Isturisa, osilodrostat [a]	CS	1.36	Oral
Zeposia, ozanimod [b]	RRMS	3.44	Oral
Koselugo, selumetinib [a]	Neurofibromas	3.73	Oral
Tukysa, tucatinib [b]	Advanced Breast Cancer	5.17	Oral
Pemazyre, pemigatinib [b]	Bile Duct Cancer	2.73	Oral
Ongentys, opicaone [b]	Parkinson's Disease	2.31	Oral
Tabrecta, capmatinib [b]	NSCLC	1.88	Oral
Retevmo, selpercatinib [b]	Lung Cancer	2.82	Oral
Qinlock, ripretinib [b]	Stromal Tumor	4.97	Oral
Cerianna, fluoroestradiol [b]	Breast Cancer	N/A	Parenteral
Artesunate [b]	Severe Malaria	2.93	Oral
Tauvid, flortaucipir [a]	Alzheimer's Disease	N/A	Parenteral
Zepzelca, lurbinectedin [b]	Metastatic Small Cell Lung Cancer	4.11	Parenteral
Dojolvi, triheptanoin [b]	LC-FAOD	8.38	Oral
Rukobia, fostemsavir [a]	HIV	-1.38	Oral
Byfavo, remimazolam [b]	General Anesthesia	2.81	Parenteral
Inqovi, cedazuridine [b]	Myelodysplastic Syndrome	-1.90	Oral
Xeglyze, abametapir [a]	Head Lice	2.56	Topical
Lampit, nifurtimox [b]	American Trypanosomiasis	0.02	Oral

<table>
<tr><td colspan="2">Olinvyk, oliceridine [b]</td><td>Acute Pain</td><td>3.47</td><td>Parenteral</td></tr>
<tr><td colspan="2">Evrysdil, risdiplam[b]</td><td>SMA</td><td>1.92</td><td>Oral</td></tr>
<tr><td colspan="2">Winlevi, clascoterone [a]</td><td>Acne And Hair Loss</td><td>4.44</td><td>Topical</td></tr>
<tr><td colspan="2">Gavreto, pralsetinb [b]</td><td>NSCLC</td><td>2.92</td><td>Oral</td></tr>
<tr><td colspan="2">Veklury, remdesivir [a]</td><td>COVID-19</td><td>2.32</td><td>Parenteral</td></tr>
<tr><td colspan="2">Zokinvy, lonafarnib [a]</td><td>Premature Aging</td><td>3.49</td><td>Oral</td></tr>
<tr><td colspan="2">Imcivree, setmelanotide [a]</td><td>Obesity</td><td>-1.89</td><td>Parenteral</td></tr>
<tr><td colspan="2">Psma-11,ga 68 psma-11 [a]</td><td>Detection And Localization Of Pca</td><td>-3.50</td><td>Parenteral</td></tr>
<tr><td colspan="2">Orladeyo, berotralstat [b]</td><td>Hereditary Angioedema</td><td>4.58</td><td>Oral</td></tr>
<tr><td colspan="2">Klisyri, tirbanibulin [a]</td><td>Actinic Keratosis</td><td>3.25</td><td>Topical</td></tr>
<tr><td colspan="2">Orgovyx, relugolix [b]</td><td>Prostate Cancer</td><td>4.60</td><td>Oral</td></tr>
<tr><td colspan="2">Gemtesa, vibegron [b]</td><td>Overactive Bladder</td><td>1.87</td><td>Oral</td></tr>
<tr><th colspan="2">Drug Name
(Trade Name, Original Name)
2021</th><th>Indications</th><th>ClogP</th><th>Route of administration</th></tr>
<tr><td colspan="2">Vericiguat, verquvo [a]</td><td>Heart Failure</td><td>0.27</td><td>Oral</td></tr>
<tr><td rowspan="2">Cabenuva [b]</td><td>cabotegravir</td><td>HIV</td><td>-0.15</td><td>Parenteral</td></tr>
<tr><td>rilpivirine</td><td>HIV</td><td>5.45</td><td>Parenteral</td></tr>
<tr><td colspan="2">Tepotinib, tepmetko [b]</td><td>NSCLC</td><td>3.40</td><td>Oral</td></tr>
<tr><td colspan="2">Umbralisib, ukoniq [b]</td><td>Marginal Zone Lymphoma</td><td>5.56</td><td>Oral</td></tr>
<tr><td colspan="2">Trilacicilib, cosela [a]</td><td>SCLC</td><td>3.48</td><td>Parenteral</td></tr>
</table>

Fosdenopterin, nulibry [a]		MoCD	-5.69	Parenteral
Azstarys [a]	serdexmethyl phenidate	ADHD	-1.53	Oral
	dexmethylph enidate	ADHD	2.56	Oral
Tivozanib, fotivda [b]		RCC	4.62	Oral
Ponesimod, ponvory [b]		Multiple Sclerosis	4.82	Oral
Viloxazine, qelbree [b]		ADHD	1.76	Oral
Drospirenone nextstellis [b]		Oral Contraceptives	2.84	Oral
Sotorasib, lumarkras [b]		KRAS G12CNSCL	6.16	Oral
Infigratinib, truseltiq [b]		Cholangiocarci nom	5.64	Oral
Samidorphan, lybalvi [b]		BPD	1.00	Oral
Ibrexafungerp, brexafemme [a]		Mycotic Vaginitis	5.77	Oral/Parenteral
Finerenone, kerendia [a]		CDK	4.11	Oral
Fexinidazole, fexinidazole [a]		HAT	2.38	Oral
Belumosudil, rezurock [a]		cGVHD	5.02	Oral
Odevixibat, bylvay [a]		PFIC	7.75	Oral
Belzutifan, welireg [a]		ccRCC	1.42	Oral
Difelikefalin, korsuva [a]		CKD	-0.28	Parenteral
Mobocertinib, exkivity [b]		NSCLC	5.48	Parenteral
Atogepant, qulipta [b]		Migraine Prevention	3.28	Oral
Maralixibat, livmarli [b]		ALGS	6.88	Oral
**Drug Name**		**Indications**	**ClogP**	**Route of**

(Trade Name, Original Name)<br>2022			administration
Quviviq, daridorexant [b]	Insomnia	6.02	Oral
Cibinqo, abrocitinib [b]	Atopic Dermatitis	2.36	Oral
Pyrukynd, mitapivat [a]	Hemolytic Anemia	3.44	Oral
Vonjo, pacritinib [b]	Myelofibrosis	2.46	Oral
Ztalmy, ganaxolone [b]	CDKL5 deficiency (CDD)	5.03	Oral/Parenteral
Vivjoa, oteseconazole [b]	RVVC	3.67	Oral
Camzyos, mavacamten [a]	HCM	2.42	Oral
Voquezna, vonoprazan[a]	Helicobacter Pylori Infection	2.54	Oral
Vtama, tapinarof [a]	Plaque Psoriasis	4.53	Topical
Sotyktu, deucravacitin11b [a]	Plaque Psoriasis	1.88	Oral
Omlonti, omidenepag isopropyl [b]	Glaucoma	3.04	Topical
Relyvrio, sodium phenylbutyrate taurursodiol [b]	ALS	1.68	Oral
Lytgobi, futibatinib [b]	Intrahepatic Bile Duct Carcinoma	1.96	Oral
Rezlidhia, olutasidenib [b]	R/R AML	1.11	Oral
Krazati, adagrasib [b]	NSCLC	5.82	Oral
Sunlenca, lenacapavir [b]	HIV-1	1.98	Oral/Parenteral

Supplementary Table S3. The Canonical SMILES of small molecule drugs approved by the FDA

Drug Name (Trade Name, Original Name) 2012	Canonical SMILES
Orkambi, ivacaftor [b]	CC1=C(N=C(C=C1)NC(=O)C2(CC2)C3=CC4=C(C=C3)OC(O4)(F)F)C5=CC(=CC=C5)C(=O)O.CC(C)(C)C1=CC(=C(C=C1NC(=O)C2=CNC3=CC=CC=C3C2=O)O)C(C)(C)C
Xeljanz, tofacitinib [b]	CC1CCN(CC1N(C)C2=NC=NC3=C2C=CN3)C(=O)CC#N.C(C(=O)O)C(CC(=O)O)(C(=O)O)O
Xtandi, enzalutami [b]	CC1(C(=O)N(C(=S)N1C2=CC(=C(C=C2)C(=O)NC)F)C3=CC(=C(C=C3)C#N)C(F)(F)F)C
Eliquis, apixaban [b]	COC1=CC=C(C=C1)N2C3=C(CCN(C3=O)C4=CC=C(C=C4)N5CCCCC5=O)C(=N2)C(=O)N
Picato, ingenol mebutate [a]	CC=C(C)C(=O)OC1C(=CC23C1(C(C(=CC(C2=O)C4C(C4(C)C)CC3C)CO)O)O)C
Inlyta, axitinib [b]	CNC(=O)C1=CC=CC=C1SC2=CC3=C(C=C2)C(=NN3)C=CC4=CC=CC=N4
Erivedge, vismodegib [b]	CS(=O)(=O)C1=CC(=C(C=C1)C(=O)NC2=CC(=C(C=C2)Cl)C3=CC=CC=N3)Cl
Zioptan, tafluprost [b]	CC(C)OC(=O)CCCC=CCC1C(CC(C1C=CC(COC2=CC=CC=C2)(F)F)O)O
Stendra, avanafil [b]	COC1=C(C=C(C=C1)CNC2=NC(=NC=C2C(=O)NCC3=NC=CC=N3)N4CCCC4CO)Cl
Belviq, lorcaserin [b]	CC1CNCCC2=C1C=C(C=C2)Cl
Mybetriq, mirabegron [a]	C1=CC=C(C=C1)C(CNCCC2=CC=C(C=C2)NC(=

	O)CC3=CSC(=N3)N)O
Kyprolis, carfilzomib [b]	CC(C)CC(C(=O)C1(CO1)C)NC(=O)C(CC2=CC=CC=C2)NC(=O)C(CC(C)C)NC(=O)C(CCC3=CC=CC=C3)NC(=O)CN4CCOCC4
Tudorza pressair, aclidinum [b]	C1C[N+]2(CCC1C(C2)OC(=O)C(C3=CC=CS3)(C4=CC=CS4)O)CCCOC5=CC=CC=C5.[Br-]
Bosulif, bosutinib [b]	CN1CCN(CC1)CCCOC2=C(C=C3C(=C2)N=CC(=C3NC4=CC(=C(C=C4Cl)Cl)OC)C#N)OC
Aubagio, teriflunomid [b]	CC(=C(C#N)C(=O)NC1=CC=C(C=C1)C(F)(F)F)O
Stivaga, regorafenib [b]	CNC(=O)C1=NC=CC(=C1)OC2=CC(=C(C=C2)NC(=O)NC3=CC(=C(C=C3)Cl)C(F)(F)F)F
Fycompa, perampanel [a]	C1=CC=C(C=C1)N2C=C(C=C(C2=O)C3=CC=CC=C3C#N)C4=CC=CC=N4
Cometriq, cabozantinib [b]	COC1=CC2=C(C=CN=C2C=C1OC)OC3=CC=C(C=C3)NC(=O)C4(CC4)C(=O)NC5=CC=C(C=C5)F
Lclusig, ponatinib [b]	CC1=C(C=C(C=C1)C(=O)NC2=CC(=C(C=C2)CN3CCN(CC3)C)C(F)(F)F)C#CC4=CN=C5N4N=CC=C5
Juxtapid, lomitapide [a]	C1CN(CCC1NC(=O)C2=CC=CC=C2C3=CC=C(C=C3)C(F)(F)F)CCCCC4(C5=CC=CC=C5C6=CC=CC=C64)C(=O)NCC(F)(F)F
Sirturo, bedaquiline [a]	CN(C)CCC(C1=CC=CC2=CC=CC=C21)(C(C3=CC=CC=C3)C4=C(N=C5C=CC(=CC5=C4)Br)OC)O
**Drug Name (Trade Name, Original Name) 2013**	**Canonical SMILES**

Pomalyst, pomalidomide [b]	C1CC(=O)NC(=O)C1N2C(=O)C3=C(C2=O)C(=CC=C3)N
Tafinlar, dabrafenib mesylate [b]	CC(C)(C)C1=NC(=C(S1)C2=NC(=NC=C2)N)C3=C(C(=CC=C3)NS(=O)(=O)C4=C(C=CC=C4F)F)F
Mekinist, trametinib dimethyl sulfoxide [b]	CC1=C2C(=C(N(C1=O)C)NC3=C(C=C(C=C3)I)F)C(=O)N(C(=O)N2C4=CC=CC(=C4)NC(=O)C)C5CC5
Gilotrif, afatinib dimaleate [b]	CN(C)CC=CC(=O)NC1=C(C=C2C(=C1)C(=NC=N2)NC3=CC(=C(C=C3)F)Cl)OC4CCOC4.C(=CC(=O)O)C(=O)O.C(=CC(=O)O)C(=O)O
Imbruvica, ibrutinib [a]	C=CC(=O)N1CCCC(C1)N2C3=NC=NC(=C3C(=N2)C4=CC=C(C=C4)OC5=CC=CC=C5)N
Tivicay, dolutegravir sodium [b]	CC1CCOC2N1C(=O)C3=C(C(=O)C(=CN3C2)C(=O)NCC4=C(C=C(C=C4)F)F)O
Olysio, simeprevir [b]	CC1=C(C=CC2=C1N=C(C=C2OC3CC4C(C3)C(=O)N(CCCCC=CC5CC5(NC4=O)C(=O)NS(=O)(=O)C6CC6)C)C7=NC(=CS7)C(C)C)OC
Sovaldi, sofosbuvir [a]	CC(C)OC(=O)C(C)NP(=O)(OCC1C(C(C(O1)N2C=CC(=O)NC2=O)(C)F)O)OC3=CC=CC=C3
Nesina, alogliptin benzoate [b]	CN1C(=O)C=C(N(C1=O)CC2=CC=CC=C2C#N)N3CCCC(C3)N.C1=CC=C(C=C1)C(=O)O
Invokana, canagliflozin [a]	CC1=C(C=C(C=C1)C2C(C(C(C(O2)CO)O)O)O)CC3=CC=C(S3)C4=CC=C(C=C4)F
Adempas, riociguat [a]	CN(C1=C(N=C(N=C1N)C2=NN(C3=C2C=CC=N3)CC4=CC=CC=C4F)N)C(=O)OC
Opsumit, macitentan [b]	CCCNS(=O)(=O)NC1=C(C(=NC=N1)OCCOC2=NC=C(C=N2)Br)C3=CC=C(C=C3)Br
Breo ellipta, vilanterol trifenatate [b]	CC1CC2C3CC(C4=CC(=O)C=CC4(C3(C(CC2(C1(C(=O)SCF)OC(=O)C5=CC=CO5)C)O)F)C)F.C1=

		CC(=C(C(=C1)Cl)COCCOCCCCCCNCC(C2=CC(=C(C=C2)O)CO)O)Cl
Bromide, umeclidinium [b]		[Br-]
Osphena, ospemifene [b]		C1=CC=C(C=C1)C(=C(C2=CC=CC=C2)C3=CC=C(C=C3)OCCO)CCCl
Tecfidera, dimethyl fumarate [a]		COC(=O)C=CC(=O)OC
Brintellix, vortioxetine hydrobromide [b]		CC1=CC(=C(C=C1)SC2=CC=CC=C2N3CCNCC3)C.Br
Aotiom, eslicarbazepine acctate [b]		C1C(C2=CC=CC=C2N(C3=CC=CC=C31)C(=O)N)O
Luzu, luliconazole [b]		C1C(SC(=C(C#N)N2C=CN=C2)S1)C3=C(C=C(C=C3)Cl)Cl
**Drug Name (Trade Name, Original Name) 2014**		**Canonical SMILES**
Zykadia, ceritinib [b]		CC1=CC(=C(C=C1C2CCNCC2)OC(C)C)NC3=NC=C(C(=N3)NC4=CC=CC=C4S(=O)(=O)C(C)C)Cl
Beleodaq, belinostat [b]		C1=CC=C(C=C1)NS(=O)(=O)C2=CC=CC(=C2)C=CC(=O)NO
Zydelig, idelaisib [a]		CCC(C1=NC2=C(C(=CC=C2)F)C(=O)N1C3=CC=CC=C3)NC4=NC=NC5=C4NC=N5
Ledipasvir [b]		CC(C)C(C(=O)N1CC2(CC2)CC1C3=NC=C(N3)C4=CC5=C(C=C4)C6=C(C5(F)F)C=C(C=C6)C7=CC8=C(C=C7)N=C(N8)C9C1CCC(C1)N9C(=O)C(C(C)C)NC(=O)OC)NC(=O)OC
Rapivab, peramivir [b]		CCC(CC)C(C1C(CC(C1O)C(=O)O)N=C(N)N)NC(=O)C
Viekira pak[b]	ombitasvir	CC(C)C(C(=O)N1CCCC1C(=O)NC2=CC=C(C=C

		2)C3CCC(N3C4=CC=C(C=C4)C(C)(C)C)C5=CC=C(C=C5)NC(=O)C6CCCN6C(=O)C(C(C)C)NC(=O)OC)NC(=O)OC
	paritaprevir	CC1=CN=C(C=N1)C(=O)NC2CCCCCC=CC3CC3(NC(=O)C4CC(CN4C2=O)OC5=NC6=CC=CC=C6C7=CC=CC=C75)C(=O)NS(=O)(=O)C8CC8
	dasabuvir	CC(C)(C)C1=CC(=CC(=C1OC)C2=CC3=C(C=C2)C=C(C=C3)NS(=O)(=O)C)N4C=CC(=O)NC4=O
Impavido, miltefosine [a]		CCCCCCCCCCCCCCCCOP(=O)([O-])OCC[N+](C)(C)C
Farxiga, dapagliflozin [b]		CCOC1=CC=C(C=C1)CC2=C(C=CC(=C2)C3C(C(C(C(O3)CO)O)O)O)Cl
Jardiance, empagliflozin [b]		C1COCC1OC2=CC=C(C=C2)CC3=C(C=CC(=C3)C4C(C(C(C(O4)CO)O)O)O)Cl
Northera, droxidopa [a]		C1=CC(=C(C=C1C(C(C(=O)O)N)O)O)O
Hetlioz, tasimelteom [b]		CCC(=O)NCC1CC1C2=C3CCOC3=CC=C2
Belsomra, suvorexant [a]		CC1CCN(CCN1C(=O)C2=C(C=CC(=C2)C)N3N=CC=N3)C4=NC5=C(O4)C=CC(=C5)Cl
Cerdelga, eliglustat tartrate [b]		CCCCCCCC(=O)NC(CN1CCCC1)C(C2=CC3=C(C=C2)OCCO3)O.CCCCCCCC(=O)NC(CN1CCCC1)C(C2=CC3=C(C=C2)OCCO3)O.C(C(C(=O)O)O)(C(=O)O)O
Movantik, naloxegol oxalate [b]		COCCOCCOCCOCCOCCOCCOCCOC1CCC2(C3CC4=C5C2(C1OC5=C(C=C4)O)CCN3CC=C)O.C(=O)(C(=O)O)O
Striverdi respimat, olodaterol hydrochloride [b]		CC(C)(CC1=CC=C(C=C1)OC)NCC(C2=C3C(=CC(=C2)O)NC(=O)CO3)O.Cl
Sivextro, tedizolid phosphate [b]		CN1N=C(N=N1)C2=NC=C(C=C2)C3=C(C=C(C=C3)N4CC(OC4=O)COP(=O)(O)O)F

Xtoro, finafloxacin [b]	C1CC1N2C=C(C(=O)C3=CC(=C(C(=C32)C#N)N4CC5C(C4)OCCN5)F)C(=O)O
Zerbaxa, ceftolozane [b]	CC(C)(C(=O)O)ON=C(C1=NSC(=N1)N)C(=O)NC2C3N(C2=O)C(=C(CS3)C[N+]4=CC(=C(N4C)N)NC(=O)NCCN)C(=O)[O-]
Dalvance, dalbavancin hydrochloride [b]	CC(C)CCCCCCCCC(=O)NC1C(C(C(OC1OC2=C3C=C4C=C2OC5=C(C=C(C=C5)C(C6C(=O)NC(C7=C(C(=CC(=C7)O)OC8C(C(C(C(O8)CO)O)O)O)O)C9=C(C=CC(=C9)C(C(=O)N6)NC(=O)C4NC(=O)C1C2=C(C(=CC(=C2)OC2=C(C=CC(=C2)C(C(=O)NC(CC2=CC=C(O3)C=C2)C(=O)N1)NC)O)O)Cl)O)C(=O)NCCCN(C)C)O)Cl)C(=O)O)O)O.Cl
Jublia, efinaconazole [b]	CC(C(CN1C=NC=N1)(C2=C(C=C(C=C2)F)F)O)N3CCC(=C)CC3
Kerydin, tavaborole [a]	B1(C2=C(CO1)C=C(C=C2)F)O
Otezla, apremilast [a]	CCOC1=C(C=CC(=C1)C(CS(=O)(=O)C)N2C(=O)C3=C(C2=O)C(=CC=C3)NC(=O)C)OC
Zontivity, vorapaxar sulfate [a]	CCOC(=O)NC1CCC2C(C1)CC3C(C2C=CC4=NC=C(C=C4)C5=CC(=CC=C5)F)C(OC3=O)C.OS(=O)(=O)O
Akynzeo, netupitant [b]	CC1=CC=CC=C1C2=CC(=NC=C2N(C)C(=O)C(C)(C)C3=CC(=CC(=C3)C(F)(F)F)C(F)(F)F)N4CCN(CC4)C
Esbrie, pirfenidone [b]	CC1=CN(C(=O)C=C1)C2=CC=CC=C2
Ofev, nintedanib [a]	CCS(=O)(=O)O.CN1CCN(CC1)CC(=O)N(C)C2=CC=C(C=C2)N=C(C3=CC=CC=C3)C4=C(NC5=C4C=CC(=C5)C(=O)OC)O
**Drug Name (Trade Name, Original Name)**	**Canonical SMILES**

2015	
Ibrance, palbociclib [a]	CC1=C(C(=O)N(C2=NC(=NC=C12)NC3=NC=C(C=C3)N4CCNCC4)C5CCCC5)C(=O)C
Lenvima, lenvatinib [b]	COC1=CC2=NC=CC(=C2C=C1C(=O)N)OC3=CC(=C(C=C3)NC(=O)NC4CC4)Cl.CS(=O)(=O)O
Fardak, panobinostat [b]	CC1=C(C2=CC=CC=C2N1)CCNCC3=CC=C(C=C3)C=CC(=O)NO
Odomzo, sonidegib [b]	CC1CN(CC(O1)C)C2=NC=C(C=C2)NC(=O)C3=CC=CC(=C3C)C4=CC=C(C=C4)OC(F)(F)F
Lonsurf, trifluridine [b]	C1C(C(OC1N2C=C(C(=O)NC2=O)C(F)(F)F)CO)O
Cotellic, trifluridine[b]	C1C(C(OC1N2C=C(C(=O)NC2=O)C(F)(F)F)CO)O
Ninlaro, ixazomib [b]	B1(OC(=O)C(O1)(CC(=O)O)CC(=O)O)C(CC(C)C)NC(=O)CNC(=O)C2=C(C=CC(=C2)Cl)Cl
Yonndelis, trabectedin [b]	CC1=CC2=C(C3C4C5C6=C(C(=C7C(=C6C(N4C(C(C2)N3C)O)COC(=O)C8(CS5)C9=CC(=C(C=C9CCN8)O)OC)OCO7)C)OC(=O)C)C(=C1OC)O
Tagrisso, osimertinib [b]	CN1C=C(C2=CC=CC=C21)C3=NC(=NC=C3)NC4=C(C=C(C(=C4)NC(=O)C=C)N(C)CCN(C)C)OC
Alecensa, alectinib [b]	CCC1=CC2=C(C=C1N3CCC(CC3)N4CCOCC4)C(C5=C(C2=O)C6=C(N5)C=C(C=C6)C#N)(C)C
Corlanor, ivabradine [a]	CN(CCCN1CCC2=CC(=C(C=C2CC1=O)OC)OC)CC3CC4=CC(=C(C=C34)OC)OC
Savaysa, edoxaban [b]	CN1CCC2=C(C1)SC(=N2)C(=O)NC3CC(CCC3NC(=O)C(=O)NC4=NC=C(C=C4)Cl)C(=O)N(C)C
Kengreal, cangrelor [b]	CSCCNC1=C2C(=NC(=N1)SCCC(F)(F)F)N(C=N2)C3C(C(C(O3)COP(=O)(O)OP(=O)(C(P(=O)(O)

		O)(Cl)Cl)O)O)O
Entresto, sacubitril [a]		CCOC(=O)C(C)CC(CC1=CC=C(C=C1)C2=CC=CC=C2)NC(=O)CCC(=O)O
Veltassa, patiromer [b]		N/A
Uptravi, selexipag [b]		CC(C)N(CCCCOCC(=O)NS(=O)(=O)C)C1=CN=C(C(=N1)C2=CC=CC=C2)C3=CC=CC=C3
Rexulti, brexpiprazole [b]		C1CN(CCN1CCCCOC2=CC3=C(C=C2)C=CC(=O)N3)C4=C5C=CSC5=CC=C4
Vraylar, cariprazine [b]		CN(C)C(=O)NC1CCC(CC1)CCN2CCN(CC2)C3=C(C(=CC=C3)Cl)Cl.Cl
Aristada, aripiprazole lauroxil [b]		CCCCCCCCCCCC(=O)OCN1C(=O)CCC2=C1C=C(C=C2)OCCCCN3CCN(CC3)C4=C(C(=CC=C4)Cl)Cl
Daklinza, daclatasvir [b]		CC(C)C(C(=O)N1CCCC1C2=NC=C(N2)C3=CC=C(C=C3)C4=CC=C(C=C4)C5=CN=C(N5)C6CCCN6C(=O)C(C(C)C)NC(=O)OC)NC(=O)OC
Genvoya [b]	cobicistat	CC(C)C1=NC(=CS1)CN(C)C(=O)NC(CCN2CCOCC2)C(=O)NC(CCC(CC3=CC=CC=C3)NC(=O)OCC4=CN=CS4)CC5=CC=CC=C5
	tenofovir alafenamide fumarate	CC(C)OC(=O)C(C)NP(=O)(COC(C)CN1C=NC2=C(N=CN=C21)N)OC3=CC=CC=C3.CC(C)OC(=O)C(C)NP(=O)(COC(C)CN1C=NC2=C(N=CN=C21)N)OC3=CC=CC=C3.C(=CC(=O)O)C(=O)O
	emtricitabine	C1C(OC(S1)CO)N2C=C(C(=NC2=O)N)F
	elvitegravir	CC(C)C(CO)N1C=C(C(=O)C2=C1C=C(C(=C2)CC3=C(C(=CC=C3)Cl)F)OC)C(=O)O
Avycaz, avibactam sodium [b]		CC(C)(C(=O)O)ON=C(C1=CSC(=N1)N)C(=O)NC2C3N(C2=O)C(=C(CS3)C[N+]4=CC=CC=C4)C(=O)[O-].C1CC(N2CC1N(C2=O)OS(=O)(=O)O)C

	(=O)N
Cresemba, isavuconazoium sulfate [b]	CC(C1=NC(=CS1)C2=CC=C(C=C2)C#N)C(CN3C=[N+](C=N3)C(C)OC(=O)N(C)C4=C(C=CC=N4)COC(=O)CNC)(C5=C(C=CC(=C5)F)F)O.OS(=O)(=O)[O-]
Cholbam, cholic acid [b]	CC(CCC(=O)O)C1CCC2C1(C(CC3C2C(CC4C3(CCC(C4)O)C)O)O)C
Kybella, deoxycholic acid [b]	CC(CCC(=O)O)C1CCC2C1(C(CC3C2CCC4C3(CCC(C4)O)C)O)C
Varubi, rolapitant [b]	CC(C1=CC(=CC(=C1)C(F)(F)F)C(F)(F)F)OCC2(CCC3(CCC(=O)N3)CN2)C4=CC=CC=C4
Xuriden, uridine triacetate [a]	CC(=O)OCC1C(C(C(O1)N2C=CC(=O)NC2=O)OC(=O)C)OC(=O)C
Orkambi, lumacaftor [a]	CC1=C(N=C(C=C1)NC(=O)C2(CC2)C3=CC4=C(C=C3)OC(O4)(F)F)C5=CC(=CC=C5)C(=O)O
Zurampic, lesinurad [b]	C1CC1C2=CC=C(C3=CC=CC=C23)N4C(=NN=C4Br)SCC(=O)O
Addyi, flibanserin [a]	C1CN(CCN1CCN2C3=CC=CC=C3NC2=O)C4=CC=CC(=C4)C(F)(F)F
Bridion, sugammadex [a]	C(CSCC1C2C(C(C(O1)OC3C(OC(C(C3O)O)OC4C(OC(C(C4O)O)OC5C(OC(C(C5O)O)OC6C(OC(C(C6O)O)OC7C(OC(C(C7O)O)OC8C(OC(C(C8O)O)OC9C(OC(O2)C(C9O)O)CSCCC(=O)O)CSCCC(=O)O)CSCCC(=O)O)CSCCC(=O)O)CSCCC(=O)O)CSCCC(=O)O)O)O)C(=O)O
Viberzi, eluxadoline [b]	CC1=CC(=CC(=C1CC(C(=O)N(CC2=CC(=C(C=C2)OC)C(=O)O)C(C)C3=NC=C(N3)C4=CC=CC=C4)N)C)C(=O)N
**Drug Name**	**Canonical SMILES**

(Trade Name, Original Name) 2016		
Zepatier [b]	elbasvir	CC(C)C(C(=O)N1CCCC1C2=NC=C(N2)C3=CC4=C(C=C3)N5C(OC6=C(C5=C4)C=CC(=C6)C7=CN=C(N7)C8CCCN8C(=O)C(C(C)C)NC(=O)OC)C9=CC=CC=C9)NC(=O)OC
	grazoprevir	CC(C)(C)C1C(=O)N2CC(CC2C(=O)NC3(CC3C=C)C(=O)NS(=O)(=O)C4CC4)OC5=NC6=C(C=CC(=C6)OC)N=C5CCCCCC7CC7OC(=O)N1
Epclusa, brivaracetam [b]		CC1CCC(N1C(=O)C(C(C)C)NC(=O)OC)C2=NC3=C(N2)C=CC4=CC5=C(C=C43)OCC6=C5C=CC(=C6)C7=CN=C(N7)C8CC(CN8C(=O)C(C9=CC=CC=C9)NC(=O)OC)COC.CC(C)OC(=O)C(C)NP(=O)(OCC1C(C(C(O1)N2C=CC(=O)NC2=O)(C)F)O)OC3=CC=CC=C3
Briviact, brivaracetam [b]		CCCC1CC(=O)N(C1)C(CC)C(=O)N
Nuplazid, pimavanserin [b]		CC(C)COC1=CC=C(C=C1)CNC(=O)N(CC2=CC=C(C=C2)F)C3CCN(CC3)C.CC(C)COC1=CC=C(C=C1)CNC(=O)N(CC2=CC=C(C=C2)F)C3CCN(CC3)C.C(C(C(=O)O)O)(C(=O)O)O
Eucrisa, crisaborole [b]		B1(C2=C(CO1)C=C(C=C2)OC3=CC=C(C=C3)C#N)O
Rubraca, rucaparib [b]		CNCC1=CC=C(C=C1)C2=C3CCNC(=O)C4=C3C(=CC(=C4)F)N2
Venclexta, venetoclax [a]		CC1(CCC(=C(C1)C2=CC=C(C=C2)Cl)CN3CCN(CC3)C4=CC(=C(C=C4)C(=O)NS(=O)(=O)C5=CC(=C(C=C5)NCC6CCOCC6)[N+](=O)[O-])OC7=CN=C8C(=C7)C=CN8)C
Ocaliva, obeticholic acid [a]		CCC1C2CC(CCC2(C3CCC4(C(C3C1O)CCC4C(C

		)CCC(=O)O)C)C)O
Xiidra, benznidazol [a]		CS(=O)(=O)C1=CC=CC(=C1)CC(C(=O)O)NC(=O)C2=C(C=C3CN(CCC3=C2Cl)C(=O)C4=CC5=C(C=C4)C=CO5)Cl
**Drug Name (Trade Name, Original Name) 2017**		**Canonical SMILES**
Baxdela, delafloxacin [b]		C1C(CN1C2=C(C=C3C(=C2Cl)N(C=C(C3=O)C(=O)O)C4=C(C=C(C(=N4)N)F)F)F)O
Vabomere, secnidazole [b]		B1(C(CCC(O1)CC(=O)O)NC(=O)CC2=CC=CS2)O.CC1C2C(C(=O)N2C(=C1SC3CC(NC3)C(=O)N(C)C)C(=O)O)C(C)O
Solosec, secnidazole [b]		CC1=NC=C(N1CC(C)O)[N+](=O)[O-]
Xepi, ozenoxacin [b]		CC1=CC(=CN=C1NC)C2=C(C3=C(C=C2)C(=O)C(=CN3C4CC4)C(=O)O)C
Vosevi, voxilaprevir [b]		CCC1C2CN(C1C(=O)NC3(CC3C(F)F)C(=O)NS(=O)(=O)C4(CC4)C)C(=O)C(NC(=O)OC5CC5CCCCC(C6=NC7=C(C=C(C=C7)OC)N=C6O2)(F)F)C(C)(C)C
Mavyret [b]	glecaprevir	CC1(CC1)S(=O)(=O)NC(=O)C2(CC2C(F)F)NC(=O)C3CC4CN3C(=O)C(NC(=O)OC5CCCC5OCC=CC(C6=NC7=CC=CC=C7N=C6O4)(F)F)C(C)(C)C
	pibrentasvir	CC(C(C(=O)N1CCCC1C2=NC3=C(N2)C=C(C(=C3)F)C4CCC(N4C5=CC(=C(C(=C5)F)N6CCC(CC6)C7=CC=C(C=C7)F)F)C8=CC9=C(C=C8F)N=C(N9)C1CCCN1C(=O)C(C(C)OC)NC(=O)OC)NC(=O)OC)OC
Prevymis, letermovir [a]		COC1=C(C=C(C=C1)C(F)(F)F)N2C(C3=C(C(=C

	C=C3)F)N=C2N4CCN(CC4)C5=CC(=CC=C5)OC)CC(=O)O
Kisqal, ribociclib [b]	CN(C)C(=O)C1=CC2=CN=C(N=C2N1C3CCCC3)NC4=NC=C(C=C4)N5CCNCC5
Zejula, niraparib [b]	C1CC(CNC1)C2=CC=C(C=C2)N3C=C4C=CC=C(C4=N3)C(=O)N
Alunbrig, midostaurin [b]	CC12C(C(CC(O1)N3C4=CC=CC=C4C5=C6C(=C7C8=CC=CC=C8N2C7=C53)CNC6=O)N(C)C(=O)C9=CC=CC=C9)OC
Rydapt, midostaurin [a]	CC12C(C(CC(O1)N3C4=CC=CC=C4C5=C6C(=C7C8=CC=CC=C8N2C7=C53)CNC6=O)N(C)C(=O)C9=CC=CC=C9)OC
Nerlynx, enasidenib [b]	CC(C)(CNC1=NC(=NC(=N1)C2=NC(=CC=C2)C(F)(F)F)NC3=CC(=NC=C3)C(F)(F)F)O
Idhifa, enasidenib [a]	CC(C)(CNC1=NC(=NC(=N1)C2=NC(=CC=C2)C(F)(F)F)NC3=CC(=NC=C3)C(F)(F)F)O
Aliqopa, copanlisib [b]	COC1=C(C=CC2=C3NCCN3C(=NC(=O)C4=CN=C(N=C4)N)N=C21)OCCCN5CCOCC5
Verzenio, abemaciclib [b]	CCN1CCN(CC1)CC2=CN=C(C=C2)NC3=NC=C(C(=N3)C4=CC5=C(C(=C4)F)N=C(N5C(C)C)C)F
Calquence, acalabrutinib [b]	CC#CC(=O)N1CCCC1C2=NC(=C3N2C=CN=C3N)C4=CC=C(C=C4)C(=O)NC5=CC=CC=N5
Bevyxxa, safinamide [b]	CN(C)C(=N)C1=CC=C(C=C1)C(=O)NC2=C(C=C(C=C2)OC)C(=O)NC3=NC=C(C=C3)Cl
Xadago, safinamide [b]	CC(C(=O)N)NCC1=CC=C(C=C1)OCC2=CC(=CC=C2)F
Benznidazole [b]	C1=CC=C(C=C1)CNC(=O)CN2C=CN=C2[N+](=O)[O-]
Steglatro, ertugliflozin [b]	CCOC1=CC=C(C=C1)CC2=C(C=CC(=C2)C34C(

		C(C(C(O3)(CO4)CO)O)O)O)Cl
Xermelo, telotristat ethyl [a]		CCOC(=O)C(CC1=CC=C(C=C1)C2=CC(=NC(=N2)N)OC(C3=C(C=C(C=C3)Cl)N4C=CC(=N4)C)C(F)(F)F)N
Emflaza, deflazacort [a]		CC1=NC2(C(O1)CC3C2(CC(C4C3CCC5=CC(=O)C=CC45C)O)C)C(=O)COC(=O)C
Symproic, naldemedine [b]		CC1=CC=C(C=C1)S(=O)(=O)O.CC(C)(C1=NC(=NO1)C2=CC=CC=C2)NC(=O)C3=C(C4C56CCN(C(C5(C3)O)CC7=C6C(=C(C=C7)O)O4)CC8CC8)O
Austedo, deutetrabenazine [b]		CC(C)CC1CN2CCC3=CC(=C(C=C3C2CC1=O)OC)OC
Ingrezza, valbenazine [b]		CC(C)CC1CN2CCC3=CC(=C(C=C3C2CC1OC(=O)C(C(C)C)N)OC)OC
Radicava, edaravone [a]		CC1=NN(C(=O)C1)C2=CC=CC=C2
Vyzulta, latanoprostene bunod [b]		C1C(C(C(C1O)CC=CCCCC(=O)OCCCCO[N+](=O)[O-])CCC(CCC2=CC=CC=C2)O)O
Rhopressa, netarsudil [a]		CC1=CC(=C(C=C1)C(=O)OCC2=CC=C(C=C2)C(CN)C(=O)NC3=CC4=C(C=C3)C=NC=C4)C
Macrilen, macimorelin acetate [a]		CC(=O)O.CC(C)(C(=O)NC(CC1=CNC2=CC=CC=C21)C(=O)NC(CC3=CNC4=CC=CC=C43)NC=O)N
**Drug Name (Trade Name, Original Name) 2018**		**Canonical SMILES**
Biktarvy [a]	bictegravir	C1CC2CC1N3C(O2)CN4C=C(C(=O)C(=C4C3=O)O)C(=O)NCC5=C(C=C(C=C5F)F)F
	emtricitabine	C1C(OC(S1)CO)N2C=C(C(=NC2=O)N)F
	TAF	CC(C)OC(=O)C(C)NP(=O)(COC(C)CN1C=NC2=

		C(N=CN=C21)N)OC3=CC=CC=C3
Symdeko [a]	tezacaftor	CC(C)(CO)C1=CC2=CC(=C(C=C2N1CC(CO)O)F)NC(=O)C3(CC3)C4=CC5=C(C=C4)OC(O5)(F)F
	ivacaftor	CC(C)(C)C1=CC(=C(C=C1NC(=O)C2=CNC3=CC=CC=C3C2=O)O)C(C)(C)C
Erleada, apalutamide [a]		CNC(=O)C1=C(C=C(C=C1)N2C(=S)N(C(=O)C23CCC3)C4=CC(=C(N=C4)C#N)C(F)(F)F)F
Tavalisse, fostamatinib [a]		CC1(C(=O)N(C2=C(O1)C=CC(=N2)NC3=NC(=NC=C3F)NC4=CC(=C(C(=C4)OC)OC)OC)COP(=O)(O)O)C
Cakynzeo, fosnetupitant [b]		CC1=CC=CC=C1C2=CC(=NC=C2N(C)C(=O)C(C)(C)C3=CC(=CC(=C3)C(F)(F)F)C(F)(F)F)N4CC[N+](CC4)(C)COP(=O)(O)[O-]
Lucemyra, lofexidine [a]		CC(C1=NCCN1)OC2=C(C=CC=C2Cl)Cl
Doptelet, avatrombopag [a]		C1CCC(CC1)N2CCN(CC2)C3=C(N=C(S3)NC(=O)C4=CC(=C(N=C4)N5CCC(CC5)C(=O)O)Cl)C6=CC(=CS6)Cl
Olumiant, baritinib [a]		CCS(=O)(=O)N1CC(C1)(CC#N)N2C=C(C=N2)C3=C4C=CNC4=NC=N3
Moxidectin [a]		CC1CC(=CCC2CC(CC3(O2)CC(=NOC)C(C(O3)C(=CC(C)C)C)C)OC(=O)C4C=C(C(C5C4(C(=CC=C1)CO5)O)O)C)C
Epidiolex, cannabidiol [a]		CCCCCC1=CC(=C(C(=C1)O)C2C=C(CCC2C(=C)C)C)O
Zemdri, plazomici [a]		CC1(COC(C(C1NC)O)OC2C(CC(C(C2O)OC3C(CC=C(O3)CNCCO)N)N)NC(=O)C(CCN)O)O
Braftiovi, encorafenib [b]		CC(C)N1C=C(C(=N1)C2=C(C(=CC(=C2)Cl)NS(=O)(=O)C)F)C3=NC(=NC=C3)NCC(C)NC(=O)OC
Mektovi, tecovirimat [a]		C1C2C1C3C=CC2C4C3C(=O)N(C4=O)NC(=O)C

	5=CC=C(C=C5)C(F)(F)F
Tpoxx, tecovirimat [a]	C1C2C1C3C=CC2C4C3C(=O)N(C4=O)NC(=O)C5=CC=C(C=C5)C(F)(F)F
Tibsovo, ivosidenib [a]	C1CC(=O)N(C1C(=O)N(C2=CC(=CN=C2)F)C(C3=CC=CC=C3Cl)C(=O)NC4CC(C4)(F)F)C5=NC=CC(=C5)C#N
Krintafel, tafenoquine [a]	CC1=CC(=NC2=C1C(=C(C=C2NC(C)CCCN)OC)OC3=CC=CC(=C3)C(F)(F)F)OC.C(CC(=O)O)C(=O)O
Orilissa, elagolix sodium [a]	CC1=C(C(=O)N(C(=O)N1CC2=C(C=CC=C2F)C(F)(F)F)CC(C3=CC=CC=C3)NCCCC(=O)O)C4=C(C(=CC=C4)OC)F
Mulpleta, lusutrombopag [a]	CCCCCCOC(C)C1=CC=CC(=C1OC)C2=CSC(=N2)NC(=O)C3=CC(=C(C(=C3)Cl)C=C(C)C(=O)O)Cl
Annovera, segesterone [a]	CC(=O)C1(C(=C)CC2C1(CCC3C2CCC4=CC(=O)CCC34)C)O
Galafold, migalastat [a]	C1C(C(C(C(N1)CO)O)O)O.Cl
Diacomit, stiripentol [a]	CC(C)(C)C(C=CC1=CC2=C(C=C1)OCO2)O
Xerava, eravacycline [a]	CN(C)C1C2CC3CC4=C(C=C(C(=C4C(=C3C(=O)C2(C(=C(C1=O)C(=O)N)O)O)O)O)NC(=O)CN5CCCC5)F.Cl.Cl
Pifeltro, doravirine [a]	CN1C(=NNC1=O)CN2C=CC(=C(C2=O)OC3=CC(=CC(=C3)C#N)Cl)C(F)(F)F
Copikra, duvelisib [b]	CC(C1=CC2=C(C(=CC=C2)Cl)C(=O)N1C3=CC=CC=C3)NC4=NC=NC5=C4NC=N5
Vizimpro, dacomitinib [a]	COC1=C(C=C2C(=C1)N=CN=C2NC3=CC(=C(C=C3)F)Cl)NC(=O)C=CCN4CCCCC4.O
Seysara, sarecycline [b]	CN(C)C1C2CC3CC4=C(C=CC(=C4C(=C3C(=O)

	C2(C(=C(C1=O)C(=O)N)O)O)O)O)CN(C)OC
Nuzyra, omadacycline [a]	CC(C)(C)CNCC1=CC(=C2CC3CC4C(C(=O)C(=C(C4(C(=O)C3=C(C2=C1O)O)O)O)O)C(=O)N)N(C)C)N(C)C
Talzenna, talazoparib [a]	CN1C(=NC=N1)C2C(NC3=CC(=CC4=C3C2=NNC4=O)F)C5=CC=C(C=C5)F
Xofluza, baloxavir marboxil [a]	COC(=O)OCOC1=C2C(=O)N3CCOCC3N(N2C=CC1=O)C4C5=C(CSC6=CC=CC=C46)C(=C(C=C5)F)F
Lorbrena, lorlatinib [a]	CC1C2=C(C=CC(=C2)F)C(=O)N(CC3=NN(C(=C3C4=CC(=C(N=C4)N)O1)C#N)C)C
Yupelri, revefenacin [a]	CN(CCN1CCC(CC1)OC(=O)NC2=CC=CC=C2C3=CC=CC=C3)C(=O)C4=CC=C(C=C4)CN5CCC(CC5)C(=O)N
Daemcolo, rifamycin [b]	CC1C=CC=C(C(=O)NC2=CC(=C3C(=C2O)C(=C(C4=C3C(=O)C(O4)(OC=CC(C(C(C(C(C(C1O)C)O)C)OC(=O)C)C)OC)C)C)O)O)C
Daurisom, glasdegib [b]	CN1CCC(CC1C2=NC3=CC=CC=C3N2)NC(=O)NC4=CC=C(C=C4)C#N
Vitrakvi, larotrectinib [a]	C1CC(N(C1)C2=NC3=C(C=NN3C=C2)NC(=O)N4CCC(C4)O)C5=C(C=CC(=C5)F)F
Firdapse, amifampridine [a]	C1=CN=CC(=C1N)N.OP(=O)(O)O
Xospata, gilteritinib [a]	CCC1=C(N=C(C(=N1)C(=O)N)NC2=CC(=C(C=C2)N3CCC(CC3)N4CCN(CC4)C)OC)NC5CCOCC5
Motegrity, prucalopride, [a]	COCCCN1CCC(CC1)NC(=O)C2=CC(=C(C3=C2OCC3)N)Cl
**Drug Name (Trade Name, Original Name)**	**Canonical SMILES**

2019	
Piqray, alpelisib [a]	CC1=C(SC(=N1)NC(=O)N2CCCC2C(=O)N)C3=CC(=NC=C3)C(C)(C)C(F)(F)F
Vyleesi, brexanolone [b]	CCCCC(C(=O)NC1CC(=O)NCCCCC(NC(=O)C(NC(=O)C(NC(=O)C(NC(=O)C(NC1=O)CC2=CN=CN2)CC3=CC=CC=C3)CCCN=C(N)N)CC4=CNC5=CC=CC=C54)C(=O)O)NC(=O)C.CC(=O)O
Zulresso, brexanolone [a]	CC(=O)C1CCC2C1(CCC3C2CCC4C3(CCC(C4)O)C)C
Rozlytrek, entrectinb [a]	CN1CCN(CC1)C2=CC(=C(C=C2)C(=O)NC3=NNC4=C3C=C(C=C4)CC5=CC(=CC(=C5)F)F)NC6CCOCC6
Balversa, erdafitinib [a]	CC(C)NCCN(C1=CC2=NC(=CN=C2C=C1)C3=CN(N=C3)C)C4=CC(=CC(=C4)OC)OC
Inrebic, fedratinib [a]	CC1=CN=C(N=C1NC2=CC(=CC=C2)S(=O)(=O)NC(C)(C)C)NC3=CC=C(C=C3)OCCN4CCCC4
Xenleta, lefamulin [a]	CC1CCC23CCC(=O)C2C1(C(CC(C(C3C)O)(C)C=C)OC(=O)CSC4CCC(CC4O)N)C
Turalio, pexidartinib [a]	C1=CC(=NC=C1CC2=CNC3=C2C=C(C=N3)Cl)NCC4=CN=C(C=C4)C(F)(F)F
Pretomanid [a]	C1C(COC2=NC(=CN21)[N+](=O)[O-])OCC3=CC=C(C=C3)OC(F)(F)F
Seysara, sarecycline hydrochloride [a]	CN(C)C1C2CC3CC4=C(C=CC(=C4C(=C3C(=O)C2(C(=C(C1=O)C(=O)N)O)O)O)O)CN(C)OC.Cl
Xpovio,selinexor [a]	C1=CN=C(C=N1)NNC(=O)C=CN2C=NC(=N2)C3=CC(=CC(=C3)C(F)(F)F)C(F)(F)F
Mayzent, siponimod [a]	CCC1=C(C=CC(=C1)C(=NOCC2=CC(=C(C=C2)C3CCCCC3)C(F)(F)F)C)CN4CC(C4)C(=O)O
Sunosi, solriamfetol [a]	C1=CC=C(C=C1)CC(COC(=O)N)N

Aklief, trifarotene [a]	CC(C)(C)C1=C(C=CC(=C1)C2=C(C=CC(=C2)C3=CC=C(C=C3)C(=O)O)OCCO)N4CCCC4
Rinvoq, upadacitinib [a]	CCC1CN(CC1C2=CN=C3N2C4=C(NC=C4)N=C3)C(=O)NCC(F)(F)F
Oxbryta, voxelotor [a]	CC(C)N1C(=CC=N1)C2=C(C=CC=N2)COC3=CC=CC(=C3C=O)O
Brukinsa, zanubrutinib [a]	C=CC(=O)N1CCC(CC1)C2CCNC3=C(C(=NN23)C4=CC=C(C=C4)OC5=CC=CC=C5)C(=O)N
**Drug Name (Trade Name, Original Name) 2020**	**Canonical SMILES**
Ayvakit, avapritinib [b]	CC(C1=CC=C(C=C1)F)(C2=CN=C(N=C2)N3CCN(CC3)C4=NC=NN5C4=CC(=C5)C6=CN(N=C6)C)N
Tazverik, tazemetostat [a]	CCN(C1CCOCC1)C2=CC(=CC(=C2C)C(=O)NCC3=C(C=C(NC3=O)C)C)C4=CC=C(C=C4)CN5CCOCC5
Nexletol, bempedoic acid [a]	CC(C)(CCCCCC(CCCCCC(C)(C)C(=O)O)O)C(=O)O
Barhemsys, amisulpride [b]	CCN1CCCC1CNC(=O)C2=CC(=C(C=C2OC)N)S(=O)(=O)CC
Nurtec odt, rimegepant [b]	C1CC(C2=C(C=CC=N2)C(C1C3=C(C(=CC=C3)F)F)N)OC(=O)N4CCC(CC4)N5C6=C(NC5=O)N=CC=C6.C1CC(C2=C(C=CC=N2)C(C1C3=C(C(=CC=C3)F)F)N)OC(=O)N4CCC(CC4)N5C6=C(NC5=O)N=CC=C6.O.O.O.OS(=O)(=O)O
Isturisa, osilodrostat [a]	C1CC2=CN=CN2C1C3=C(C=C(C=C3)C#N)F
Zeposia, ozanimod [b]	CC(C)OC1=C(C=C(C=C1)C2=NC(=NO2)C3=C4CCC(C4=CC=C3)NCCO)C#N.Cl

Koselugo, selumetinib [a]	CN1C=NC2=C1C=C(C(=C2F)NC3=C(C=C(C=C3)Br)Cl)C(=O)NOCCO
Tukysa, tucatinib [b]	CC1=C(C=CC(=C1)NC2=NC=NC3=C2C=C(C=C3)NC4=NC(CO4)(C)C)OC5=CC6=NC=NN6C=C5
Pemazyre, pemigatinib [b]	CCN1C2=C3C=C(NC3=NC=C2CN(C1=O)C4=C(C(=CC(=C4F)OC)OC)F)CN5CCOCC5
Ongentys, opicaone [b]	CC1=C(C(=[N+](C(=C1Cl)C)[O-])Cl)C2=NOC(=N2)C3=CC(=C(C(=C3)O)O)[N+](=O)[O-]
Tabrecta, capmatinib [b]	CNC(=O)C1=C(C=C(C=C1)C2=NN3C(=CN=C3N=C2)CC4=CC5=C(C=C4)N=CC=C5)F.O.Cl.Cl
Retevmo, selpercatinib [b]	CC(C)(COC1=CN2C(=C(C=N2)C#N)C(=C1)C3=CN=C(C=C3)N4CC5CC(C4)N5CC6=CN=C(C=C6)OC)O
Qinlock, ripretinib [b]	CCN1C2=CC(=NC=C2C=C(C1=O)C3=CC(=C(C=C3Br)F)NC(=O)NC4=CC=CC=C4)NC
Cerianna, fluoroestradiol [b]	CC12CCC3C(C1CC(C2O)F)CCC4=C3C=CC(=C4)O
Artesunate [b]	CC1CCC2C(C(OC3C24C1CCC(O3)(OO4)C)OC(=O)CCC(=O)O)C
Tauvid, flortaucipir [a]	C1=CC2=C(C=C1C3=CN=C(C=C3)F)NC4=C2C=NC=C4
Zepzelca, lurbinectedin [b]	CC1=CC2=C(C3C4C5C6=C(C(=C7C(=C6C(N4C(C(C2)N3C)O)COC(=O)C8(CS5)C9=C(CCN8)C2=C(N9)C=CC(=C2)OC)OCO7)C)OC(=O)C)C(=C1OC)O
Dojolvi, triheptanoin [b]	CCCCCCC(=O)OCC(COC(=O)CCCCCC)OC(=O)CCCCCC
Rukobia, fostemsavir [a]	CC1=NN(C=N1)C2=NC=C(C3=C2N(C=C3C(=O)C(=O)N4CCN(CC4)C(=O)C5=CC=CC=C5)COP(

	=O)(O)O)OC.C(C(CO)(CO)N)O
Byfavo, remimazolam [b]	CC1=CN=C2N1C3=C(C=C(C=C3)Br)C(=NC2CCC(=O)OC)C4=CC=CC=N4.C1=CC=C(C=C1)S(=O)(=O)O
Inqovi, cedazuridine [b]	C1CN(C(=O)NC1O)C2C(C(C(O2)CO)O)(F)F
Xeglyze, abametapir [a]	CC1=CN=C(C=C1)C2=NC=C(C=C2)C
Lampit, nifurtimox [b]	CC1CS(=O)(=O)CCN1N=CC2=CC=C(O2)[N+](=O)[O-]
Olinvyk, oliceridine [b]	COC1=C(SC=C1)CNCCC2(CCOC3(C2)CCCC3)C4=CC=CC=N4
Evrysdil, risdiplam[b]	CC1=CC(=NN2C1=NC(=C2)C)C3=CC(=O)N4C=C(C=CC4=N3)N5CCNC6(C5)CC6
Winlevi, clascoterone [a]	CCC(=O)OC1(CCC2C1(CCC3C2CCC4=CC(=O)CCC34C)C)C(=O)CO
Gavreto, pralsetinb [b]	CC1=CC(=NN1)NC2=NC(=NC(=C2)C)C3CCC(CC3)(C(=O)NC(C)C4=CN=C(C=C4)N5C=C(C=N5)F)OC
Veklury, remdesivir [a]	CCC(CC)COC(=O)C(C)NP(=O)(OCC1C(C(C(O1)(C#N)C2=CC=C3N2N=CN=C3N)O)O)OC4=CC=CC=C4
Zokinvy, lonafarnib [a]	C1CN(CCC1CC(=O)N2CCC(CC2)C3C4=C(CCC5=C3N=CC(=C5)Br)C=C(C=C4Br)Cl)C(=O)N
Imcivree, setmelanotide [a]	CC1C(=O)NC(C(=O)NC(C(=O)NC(C(=O)NC(C(=O)NC(CSSCC(C(=O)N1)NC(=O)C(CCCN=C(N)N)NC(=O)C)C(=O)N)CC2=CNC3=CC=CC=C32)CCCN=C(N)N)CC4=CC=CC=C4)CC5=CN=CN5.CC(=O)O.CC(=O)O.CC(=O)O
Psma-11,ga 68 psma-11 [a]	C1=CC(=C(C=C1CCC(=O)NCCCCCC(=O)NCCCCC(C(=O)O)NC(=O)NC(CCC(=O)O)C(=O)O)CN

<table>
<tr><td colspan="2"></td><td>(CCN(CC2=C(C=CC(=C2)CCC(=O)O)O)CC(=O)O)CC(=O)O)O</td></tr>
<tr><td colspan="2">Orladeyo, berotralstat [b]</td><td>C1CC1CNC(C2=CC(=C(C=C2)F)NC(=O)C3=CC(=NN3C4=CC=CC(=C4)CN)C(F)(F)F)C5=CC=CC(=C5)C#N</td></tr>
<tr><td colspan="2">Klisyri, tirbanibulin [a]</td><td>C1COCCN1CCOC2=CC=C(C=C2)C3=CN=C(C=C3)CC(=O)NCC4=CC=CC=C4</td></tr>
<tr><td colspan="2">Orgovyx, relugolix [b]</td><td>CN(C)CC1=C(SC2=C1C(=O)N(C(=O)N2CC3=C(C=CC=C3F)F)C4=NN=C(C=C4)OC)C5=CC=C(C=C5)NC(=O)NOC</td></tr>
<tr><td colspan="2">Gemtesa, vibegron [b]</td><td>C1CC(NC1CC2=CC=C(C=C2)NC(=O)C3CCC4=NC=CC(=O)N34)C(C5=CC=CC=C5)O</td></tr>
<tr><th colspan="2">Drug Name
(Trade Name, Original Name)
2021</th><th>Canonical SMILES</th></tr>
<tr><td colspan="2">Vericiguat, verquvo [a]</td><td>COC(=O)NC1=C(N=C(N=C1N)C2=NN(C3=C2C=C(C=N3)F)CC4=CC=CC=C4F)N</td></tr>
<tr><td rowspan="2">Cabenuva [b]</td><td>cabotegravir</td><td>CC1COC2N1C(=O)C3=C(C(=O)C(=CN3C2)C(=O)NCC4=C(C=C(C=C4)F)F)O</td></tr>
<tr><td>rilpivirine</td><td>CC1=CC(=CC(=C1NC2=NC(=NC=C2)NC3=CC=C(C=C3)C#N)C)C=CC#N</td></tr>
<tr><td colspan="2">Tepotinib, tepmetko [b]</td><td>CN1CCC(CC1)COC2=CN=C(N=C2)C3=CC=CC(=C3)CN4C(=O)C=CC(=N4)C5=CC=CC(=C5)C#N</td></tr>
<tr><td colspan="2">Umbralisib, ukoniq [b]</td><td>CC(C)OC1=C(C=C(C=C1)C2=NN(C3=NC=NC(=C23)N)C(C)C4=C(C(=O)C5=C(O4)C=CC(=C5)F)C6=CC(=CC=C6)F)F</td></tr>
<tr><td colspan="2">Trilacicilib, cosela [a]</td><td>CN1CCN(CC1)C2=CN=C(C=C2)NC3=NC=C4C=C5C(=O)NCC6(N5C4=N3)CCCCC6.Cl.Cl</td></tr>
</table>

Fosdenopterin, nulibry [a]		C1C2C(C(C3C(O2)NC4=C(N3)C(=O)NC(=N4)N)(O)O)OP(=O)(O1)O
Azstarys [a]	serdexmethylphenidate	COC(=O)C(C1CCCCN1C(=O)OC[N+]2=CC=CC(=C2)C(=O)NC(CO)C(=O)[O-])C3=CC=CC=C3
	dexmethylphenidate	COC(=O)C(C1CCCCN1)C2=CC=CC=C2
Tivozanib, fotivda [b]		CC1=CC(=NO1)NC(=O)NC2=C(C=C(C=C2)OC3=C4C=C(C(=CC4=NC=C3)OC)OC)Cl
Ponesimod, ponvory [b]		CCCN=C1N(C(=O)C(=CC2=CC(=C(C=C2)OCC(CO)O)Cl)S1)C3=CC=CC=C3C
Viloxazine, qelbree [b]		CCOC1=CC=CC=C1OCC2CNCCO2
Drospirenone nextstellis [b]		CC12CCC(=O)C=C1C3CC3C4C2CCC5(C4C6CC6C57CCC(=O)O7)C
Sotorasib, lumarkras [b]		CC1CN(CCN1C2=NC(=O)N(C3=NC(=C(C=C32)F)C4=C(C=CC=C4F)O)C5=C(C=CN=C5C(C)C)C)C(=O)C=C
Infigratinib, truseltiq [b]		CCN1CCN(CC1)C2=CC=C(C=C2)NC3=CC(=NC=N3)N(C)C(=O)NC4=C(C(=CC(=C4Cl)OC)OC)Cl
Samidorphan, lybalvi [b]		C1CC1CN2CCC34CC(=O)CCC3(C2CC5=C4C(=C(C=C5)C(=O)N)O)O
Ibrexafungerp, brexafemme [a]		CC(C)C(C)C1(CCC2(C3CCC4C5(COCC4(C3=CCC2(C1C(=O)O)C)CC(C5OCC(C)(C(C)(C)C)N)N6C(=NC=N6)C7=CC=NC=C7)C)C)C
Finerenone, kerendia [a]		CCOC1=NC=C(C2=C1C(C(=C(N2)C)C(=O)N)C3=C(C=C(C=C3)C#N)OC)C
Fexinidazole, fexinidazole [a]		CN1C(=CN=C1COC2=CC=C(C=C2)SC)[N+](=O)[O-]
Belumosudil, rezurock [a]		CC(C)NC(=O)COC1=CC=CC(=C1)C2=NC3=CC

	=CC=C3C(=N2)NC4=CC5=C(C=C4)NN=C5
Odevixibat, bylvay [a]	CCCCC1(CN(C2=CC(=C(C=C2S(=O)(=O)N1)OCC(=O)NC(C3=CC=C(C=C3)O)C(=O)NC(CC)C(=O)O)SC)C4=CC=CC=C4)CCCC
Belzutifan, welireg [a]	CS(=O)(=O)C1=C2C(C(C(C2=C(C=C1)OC3=CC(=CC(=C3)C#N)F)F)F)O
Difelikefalin, korsuva [a]	CC(C)CC(C(=O)NC(CCCCN)C(=O)N1CCC(CC1)(C(=O)O)N)NC(=O)C(CC2=CC=CC=C2)NC(=O)C(CC3=CC=CC=C3)N
Mobocertinib, exkivity [b]	CC(C)OC(=O)C1=CN=C(N=C1C2=CN(C3=CC=CC=C32)C)NC4=C(C=C(C(=C4)NC(=O)C=C)N(C)CCN(C)C)OC
Atogepant, qulipta [b]	CC1C(CC(C(=O)N1CC(F)(F)F)NC(=O)C2=CC3=C(CC4(C3)C5=C(NC4=O)N=CC=C5)N=C2)C6=C(C=CC(=C6F)F)F
Maralixibat, livmarli [b]	CCCCC1(CS(=O)(=O)C2=C(C=C(C=C2)N(C)C)C(C1O)C3=CC=C(C=C3)OCC4=CC=C(C=C4)C[N+]56CCN(CC5)CC6)CCCC
**Drug Name (Trade Name, Original Name) 2022**	**Canonical SMILES**
Quviviq, daridorexant [b]	CC1=C(C=CC2=C1N=C(N2)C3(CCCN3C(=O)C4=C(C=CC(=C4)OC)N5N=CC=N5)C)Cl.Cl
Cibinqo, abrocitinib [b]	CCCS(=O)(=O)NC1CC(C1)N(C)C2=NC=NC3=C2C=CN3
Pyrukynd, mitapivat [a]	C1CC1CN2CCN(CC2)C(=O)C3=CC=C(C=C3)NS(=O)(=O)C4=CC=CC5=C4N=CC=C5.C1CC1CN2CCN(CC2)C(=O)C3=CC=C(C=C3)NS(=O)(=O)C4=CC=CC5=C4N=CC=C5.O.O.O.OS(=O)(=O)O

Vonjo, pacritinib [b]	C1CCN(C1)CCOC2=C3COCC=CCOCC4=CC(=CC=C4)C5=NC(=NC=C5)NC(=C3)C=C2.C(C(=O)O)C(CC(=O)O)(C(=O)O)O
Ztalmy, ganaxolone [b]	CC(=O)C1CCC2C1(CCC3C2CCC4C3(CCC(C4)(C)O)C)C
Vivjoa, oteseconazole [b]	C1=CC(=CC=C1C2=CN=C(C=C2)C(C(CN3C=NN=N3)(C4=C(C=C(C=C4)F)F)O)(F)F)OCC(F)(F)F
Camzyos, mavacamten [a]	CC(C)N1C(=O)C=C(NC1=O)NC(C)C2=CC=CC=C2
Voquezna, vonoprazan[a]	CNCC1=CN(C(=C1)C2=CC=CC=C2F)S(=O)(=O)C3=CN=CC=C3
Vtama, tapinarof [a]	CC(C)C1=C(C=C(C=C1O)C=CC2=CC=CC=C2)O
Sotyktu, deucravacitin11b [a]	CNC(=O)C1=NN=C(C=C1NC2=CC=CC(=C2OC)C3=NN(C=N3)C)NC(=O)C4CC4
Omlonti, omidenepag isopropyl [b]	CC(C)OC(=O)CNC1=CC=CC(=N1)CN(CC2=CC=C(C=C2)N3C=CC=N3)S(=O)(=O)C4=CN=CC=C4
Relyvrio, sodium phenylbutyrate taurursodiol [b]	C1=CC=C(C=C1)CCCC(=O)[O-].[Na+]
Lytgobi, futibatinib [b]	COC1=CC(=CC(=C1)C#CC2=NN(C3=NC=NC(=C23)N)C4CCN(C4)C(=O)C=C)OC
Rezlidhia, olutasidenib [b]	CC(C1=CC2=C(C=CC(=C2)Cl)NC1=O)NC3=CC=C(N(C3=O)C)C#N
Krazati, adagrasib [b]	CN1CCCC1COC2=NC3=C(CCN(C3)C4=CC=CC5=C4C(=CC=C5)Cl)C(=N2)N6CCN(C(C6)CC#N)C(=O)C(=C)F
Sunlenca, lenacapavir [b]	CC(C)(C#CC1=NC(=C(C=C1)C2=C3C(=C(C=C2)Cl)C(=NN3CC(F)(F)F)[N-]S(=O)(=O)C)C(CC4=

	CC(=CC(=C4)F)F)NC(=O)CN5C6=C(C7CC7C6(F)F)C(=N5)C(F)(F)F)S(=O)(=O)C.[Na+]

* [a] stands for FIC small molecule drugs and [b] stands for Non-FIC drugs. The data is sourced from the FDA website *U.S. Food and Drug Administration (fda.gov)*

* The molecular weight and CLogP information for all small molecular compounds was calculated with ChemBioDraw Ultra 14.0.

* The Canonical SMILES information for all small molecular compounds was retrieved using PubChem.

* The route of administration is sourced from the FDA website *U.S. Food and Drug Administration (fda.gov)*

* N/A means the molecular weight information is not available.
